# Deficiency of circadian clock gene *Bmal1* exacerbates noncanonical inflammasome-mediated pyroptosis and lethality via Rev-erbα-C/EBPβ-SAA1 axis

**DOI:** 10.1038/s12276-024-01162-w

**Published:** 2024-02-01

**Authors:** Do-Wan Shim, Jun-Cheol Eo, Saeyoung Kim, Inhwa Hwang, BoYoung Nam, Jae-Eun Shin, Seung Hyeok Han, Je-Wook Yu

**Affiliations:** 1 Department of Microbiology and Immunology, Institute for Immunology and Immunological Diseases, Yonsei University College of Medicine, Seoul, 03722 Republic of Korea; 2 Graduate School of Medical Science, Brain Korea 21 Project, Yonsei University College of Medicine, Seoul, 03722 Republic of Korea; 3 Department of Internal Medicine, Institute of Kidney Disease Research, Yonsei University College of Medicine, Seoul, 03722 Republic of Korea

**Keywords:** Inflammasome, Circadian regulation, Immune cell death

## Abstract

Circadian arrhythmia has been linked to increased susceptibility to multiple inflammatory diseases, such as sepsis. However, it remains unclear how disruption of the circadian clock modulates molecular aspects of innate immune responses, including inflammasome signaling. Here, we examined the potential role of the circadian clock in inflammasome-mediated responses through myeloid-specific deletion of BMAL1, a master circadian clock regulator. Intriguingly, *Bmal1* deficiency significantly enhanced pyroptosis of macrophages and lethality of mice under noncanonical inflammasome-activating conditions but did not alter canonical inflammasome responses. Transcriptome analysis of enriched peritoneal myeloid cells revealed that *Bmal1* deficiency led to a marked reduction in Rev-erbα expression at steady state and a significant increase in serum amyloid A1 (SAA1) expression upon poly(I:C) stimulation. Notably, we found that the circadian regulator Rev-erbα is critical for poly(I:C)- or interferon (IFN)-β-induced SAA1 production, resulting in the circadian oscillation pattern of SAA1 expression in myeloid cells. Furthermore, exogenously applied SAA1 markedly increased noncanonical inflammasome-mediated pyroptosis of macrophages and lethality of mice. Intriguingly, our results revealed that type 1 IFN receptor signaling is needed for poly(I:C)- or IFN-β-induced SAA1 production. Downstream of the type 1 IFN receptor, Rev-erbα inhibited the IFN-β-induced association of C/EBPβ with the promoter region of *Saa1*, leading to the reduced transcription of *Saa1* in macrophages. *Bmal1*-deficient macrophages exhibited enhanced binding of C/EBPβ to *Saa1*. Consistently, the blockade of Rev-erbα by SR8278 significantly increased poly(I:C)-stimulated SAA1 transcription and noncanonical inflammasome-mediated lethality in mice. Collectively, our data demonstrate a potent suppressive effect of the circadian clock BMAL1 on the noncanonical inflammasome response via the Rev-erbα-C/EBPβ-SAA1 axis.

## BMAL1: The Circadian Regulator Dampening Inflammasome Responses

Circadian rhythms (our internal body clock controlling daily physiological cycles), regulate various biological processes. However, it’s uncertain how these rhythms influence our immune system. A recent study discovered that disruption of these rhythms in immune cells can lead to hyperactive immune responses driven by noncanonical inflammasome activation and pyroptosis, an inflammatory cell death. The researchers conducted experiments using circadian clock Bmal1-deficient mice and found that when the circadian rhythm in innate immune cells was disrupted, the mice exhibited increased pyroptosis and sepsis severity. The study suggests that maintaining a regular circadian rhythm could help regulate the immune system and prevent overactive immune responses. This study also provides a novel insight into the role of circadian clock-mediated signaling in immune response susceptibility, underscoring its significance in the treatment of inflammatory diseases.

This summary was initially drafted using artificial intelligence, then revised and fact-checked by the author.

## Introduction

Circadian rhythms are diurnal oscillations of biological processes found in all living organisms^1^. These rhythms are entrained by environmental cues known as zeitgebers, such as light, food, and temperature, which synchronize an organism’s endogenous rhythm with the Earth’s 24-h light-dark cycle^2,3^. The mammalian circadian system comprises a central clock located in the hypothalamic suprachiasmatic nucleus and peripheral clocks in all tissues and organs in the body^4,5^. These circadian clocks are regulated by autoregulatory transcription-translation feedback loops that are initiated by two core transcription factors, brain and muscle Arnt-like protein 1 (BMAL1) and circadian locomotor output cycles kaput (CLOCK)^6^. The BMAL1/CLOCK heterodimer promotes the transcription of clock-controlled genes, such as cryptochrome (*CRY*), period (*PER*), RAR-related orphan receptor alpha (*RORA*), and the nuclear receptor Rev-erbα gene (*NR1D1*), all of which regulate *Bmal1*-*Clock* transcription or protein activity^7,8^.

Interestingly, recent animal studies have revealed that circadian oscillation patterns occur in immune responses and immunological disease phenotypes^9,10^. For instance, in mice, the number of circulating Ly6C^hi^ monocytes has been found to vary depending on zeitgeber time (ZT), resulting in time-dependent fluctuations in tissue-infiltrating myeloid cells^11^. The mortality rate of mice exposed to bacteria or bacterial products varies greatly depending on the time point of exposure^12,13^. The severity of experimental autoimmune encephalomyelitis varies according to the time of the day at immunization^14^. Consistent with these mouse studies, patients with rheumatoid arthritis show the highest serum proinflammatory cytokine levels and more severe disease symptoms in the early morning^15,16^. These findings indicate that the circadian rhythm is potentially correlated with the sensitivity of the innate immune response, leading to diurnal oscillations in inflammatory disease phenotypes.

Innate immune cells such as macrophages have their own circadian rhythm, allowing fine-tuning of the immune response throughout the day^9^. *Bmal1* is a singularly essential clock gene, and its deletion significantly impairs circadian clock function^10^. Interestingly, myeloid-specific deletion of *Bmal1* enhanced the inflammatory response to bacterial infection and endotoxins in mice^11–13^. Furthermore, myeloid-specific circadian arrhythmic mice showed enhanced phenotypes of endotoxin-induced sepsis and high fat-induced hyperglycemia^11^ or atherosclerosis^17^. These findings suggest that the circadian clock in myeloid cells may serve as an internal brake to limit excessive inflammation and thus maintain homeostasis in the organism.

Nonetheless, the mechanism underlying the enhanced immune response in circadian arrhythmia remains unclear. The increase in the numbers of circulating and infiltrating monocytes in *Bmal1*-deficient mice may partly explain the phenomenon at the organismal level^11^. However, whether *Bmal1* deficiency modulates the innate immune potential of myeloid cells at the cellular and molecular levels is unclear. Furthermore, the potential involvement of the circadian clock in inflammasome- and pyroptosis-mediated responses in myeloid cells remains largely unknown. Therefore, this study aimed to investigate whether myeloid circadian rhythm disruption affects the sensitivity and intensity of the inflammasome and pyroptosis response using myeloid cell-specific *Bmal1*-deficient mice.

## Materials and methods

### Mice

C57BL/6 mice were purchased from Orient Bio and employed as wild-type (WT) mice. *Arntl*^*flox/flox*^ (JAX 007668), *Nlrp3*^–/–^ (JAX 017971), and *Gsdmd*^–*/*–^ (JAX 032410) mice were obtained from The Jackson Laboratory. *Gsdmd*^*flox/flox*^ (RBRC10762) mice were provided by RIKEN BioResource Research Center. *Ifnar1*^–/–^ mice were provided by Dr. Sung Jae Shin (Yonsei University). LysMcre mice were provided by Dr. Myung-Shik Lee (Soonchunhyang University). *Casp1*^–/–^ *Casp11*^–*/*–^ mice were provided by Dr. Nam-Hyuk Cho (Seoul National University). All genetically modified mice had a C57BL/6 background. For generation of myeloid-specific *Bmal1*-deficient mice, *Arntl*^*flox/flox*^ mice harboring a loxP-flanked neomycin-resistant cassette in exon 8 of *Arntl* were crossed with LysMcre mice. For generation of myeloid-specific *Gsdmd*^–/–^ mice, *Gsdmd*^*flox/flox*^ mice were crossed with LysMcre mice. Eight- to 12-week-old mice were used in the experiments. All mice were bred and maintained under specific pathogen-free conditions at Yonsei University College of Medicine. The animal experimental protocols were approved by the Institutional Ethical Committee of Yonsei University College of Medicine (2021-0122). All experiments were performed in accordance with the approved guidelines of the Institutional Ethical Committee.

### Mouse treatments

For induction of noncanonical inflammasome activation, mice were intraperitoneally injected with poly(I:C) (10 mg/kg) at ZT0, followed by intraperitoneal injection of lipopolysaccharide (LPS, L4391, 1 or 2 mg/kg) 7 h post-poly(I:C) injection at ZT7, according to previous studies with some modifications^18,19^. Alternatively, mice were administered a high dose of LPS (L3012, 20 mg/kg, intraperitoneal injection) at ZT4 to induce in vivo noncanonical inflammasome activation^20^. Mouse survival was monitored three times per day for 3 days.

### Reagents and antibodies

LPS (L3012, L4391), ATP, nigericin, poly(I:C), poly(dA:dT), PI, SR9009, and SR8278 were purchased from Sigma‒Aldrich. Recombinant mouse serum amyloid A1 and recombinant mouse IFN-β were obtained from R&D Systems. Flagellin was purchased from InvivoGen. LLME was purchased from Cayman. SAA1 ELISA kits were purchased from R&D Systems. The following antibodies were used in this study: anti-caspase-1 (AG-20B-0042, Adipogen), anti-NLRP3 (AG-20B-00140, Adipogen), anti-mouse IL-1β (AF-401-NA, R&D Systems), anti-caspase-11 (NB120-10454, Novus Biologicals), anti-Bmal1 (93806, Abcam), anti-GSDMD (209845, Abcam), anti-cathepsin B (AF965, R&D Systems), anti-cleaved caspase-3 (9579, Cell Signaling Technology), anti-fibrin (MVI-1007, MERU-VasImmune), anti-phospho-STAT1 (#7649, Cell Signaling Technology), anti-STAT1 (#14994, Cell Signaling Technology), anti-β-actin (sc-47778, Santa Cruz Biotechnology), anti-LAMP-1 (sc-19992, Santa Cruz Biotechnology) and anti-C/EBPβ (sc-7962, Santa Cruz Biotechnology).

### Cell culture

Mouse bone marrow cells were isolated from femurs and tibias of mice and cultured in L929-conditioned Dulbecco’s modified Eagle’s medium for 5–7 days for differentiation into BMDMs. BMDMs were maintained in L929-conditioned Dulbecco’s modified Eagle’s medium with 10% fetal bovine serum (FBS) and antibiotics. For isolation of alveolar macrophages, bronchoalveolar lavage fluid (BALF) was obtained by washing with 2% FBS-containing PBS, and alveolar macrophages were harvested by BALF through centrifugation for 10 min at 1500 RPM and 4 °C. For isolation of resident peritoneal macrophages, we collected mouse peritoneal lavage by rinsing with PBS containing 2% FBS. The peritoneal cells were then harvested by centrifugation, resuspended in RPMI 1640 media, and allowed to adhere to a cell culture dish. Following overnight incubation, the adherent cells were washed three times with warm PBS and employed as peritoneal macrophages.

### Immunoblot analysis

Cells were lysed in buffer containing 20 mM HEPES (pH 7.5), 0.5% Nonidet P-40, 50 mM KCl, 150 mM NaCl, 1.5 mM MgCl_2_, 1 mM EGTA, and protease inhibitors. Soluble lysates were fractionated by sodium dodecyl sulfate‒polyacrylamide gel electrophoresis and then transferred to polyvinylidene difluoride membranes. The membranes were stained with appropriate antibodies, developed for visualization using an enhanced chemiluminescence kit, and assessed using an image analyzer (Amersham^TM^ ImageQuant^TM^ 800). In some experiments, cell culture supernatants were precipitated with methanol/chloroform as described previously^21^ and then immunoblotted. All blots shown are representative of at least three independent experiments.

### Reverse transcription (RT)-quantitative polymerase chain reaction (qPCR)-based analysis of mRNA expression

Total RNA was isolated using TRIzol reagent (Invitrogen) or the AccuPrep^®^ Universal RNA Extraction Kit (Bioneer) and was reverse transcribed using the PrimeScript RT Master Mix Kit (TaKaRa). qPCRs were run using SYBR Premix Ex Taq (TaKaRa) and the following primers: 5′-GCC CAT CCT CTG TGA CTC AT-3′ and 5′-AGG CCA CAG GTA TTT TGT CG-3′ (mouse *Il1β*); 5′-AGT TGC CTT CTT GGG ACT GA-3′ and 5′-TCC ACG ATT TCC CAG AGA AC-3′ (mouse *Il6*); 5′-CAA TGA GCC AGA CAA CGA GG-3′ and 5′-TAC GCC AAA ATA GCT GTC GC-3′ (mouse *Arntl*); 5′-TTC CTG CTG TGC TTC TTC AC-3′ and 5′-CTT TCC ATT CAG CTG CTC CA-3′ (mouse *Ifnb*); 5′-AGC TGG TGA AGA CAT GAC GA-3′ and 5′-GGT GGG AAG TAT GTG GGA CA-3′ (mouse *Nr1d1*); 5′-ATG ACC TGC ACT CAA TGG GA-3′ and 5′-TGA GTG TTT CCT GCA AAG CC-3′ (mouse *Nr1d2*); 5′-GGA CAT GAG GAC ACC ATT GC-3′ and 5′-GTA GGA AGA AGC CCA GAC CC-3′ (mouse *Saa1*); 5′-TTC AAC CAC AGC AGC AAG AC-3′ and 5′-CTG GAT AAT GTT GCT GGG CC-3′ (mouse *Lpl*); 5′-AGC AGA ACC ACG ATA ACC CA-3′ and 5′-ATC TTG TCT GAG CTG CTG GT -3′ (mouse *Nfil3*); 5′-AGC CCT GTG AAT ACC GTG AA-3′ and 5′-TGA CTC GTT CAA TGT CCC CA-3′ (mouse *Hdc*); 5′-TCC AGG AGA GCT GTG TTT GT-3′ and 5′-CTG TGC AGT TCG AGC CTA AC-3′ (mouse *Prok2*); 5′-ACT TGG CAT CCC TGT AGC AT-3′ and 5′-GTG AAG GCC AGG AAG CAA AA-3′ (mouse *Pydc3*); 5′-CTG GAA AAC TTC ACC TGC CC-3′ and 5′-AGA CGT TCT GGG GCA TGT AA-3′ (mouse *Gcnt2*) and 5′-CGC GGT TCT ATT TTG TTG GT-3′ and 5′-AGT CGG CAT CGT TTA TGG TC-3′ (mouse *Rn18s*). The PCR conditions were 10 min at 95 °C, followed by 40 cycles of 15 sec at 95 °C and 1 min at 60-64 °C. *Rn18s* was used to normalize target gene expression to calculate changes in mRNA levels with respect to the control.

### Inflammasome activation assay

For induction of conventional NLRP3 inflammasome activation, BMDMs were primed with LPS (0.25 μg/ml, 3 h), followed by ATP treatment (2–2.5 mM, 30–45 min). For stimulation of AIM2 and NLRC4 inflammasome activation, cells were transfected with poly(dA:dT) (1 μg/ml) or flagellin (250 ng/ml) using Lipofectamine 2000 for 2 h. Inflammasome activation was determined by the presence of active caspase-1 p20 and active IL-1β in culture supernatants as evaluated by immunoblotting and by extracellular IL-1β quantification using enzyme-linked immunosorbent assays. For induction of noncanonical inflammasome activation^18,22^, cells were treated with IFN-β (500 pg/ml), followed by infection with *Escherichia coli* BL21 (MOI 20) or LPS transfection (2 μg/ml) using Lipofectamine 2000. Noncanonical inflammasome activation was assayed by the secretion of caspase-11 and the processing of GSDMD.

### Cell death assay

For quantification of pyroptosis, extracellular LDH release was measured using the CytoTox96 nonradioactive cytotoxicity assay kit (Promega) and was calculated as follows: Extracellular LDH release = [extracellular LDH/(intracellular LDH + extracellular LDH) × 100]. Cell death was also determined by quantifying PI uptake. BMDMs were seeded in black 96-well plates and incubated with 10 μg/ml PI in Opti-MEM. Subsequently, PI fluorescence was measured using a fluorescent plate reader.

### Evaluation of organ damage

After the treatments, mouse lungs were isolated and fixed in 10% formalin for 48 h. Furthermore, the lungs were embedded in paraffin, sectioned, and stained with hematoxylin and eosin. For quantification of cell death in the spleen, spleen tissues were lysed in TEN buffer (50 mM Tris-HCl, 2 mM EDTA, 150 mM NaCl, and protease inhibitors), and the soluble lysates were immunoblotted using anti-caspase-3 antibody. Changes in renal function were analyzed by measuring serum urea levels using a commercial kit. Urea was measured using a blood urea nitrogen (BUN) colorimetric detection kit (Arbor Assays).

### Enrichment of myeloid cells from peritoneal cells

For isolation of peritoneal macrophages, mouse peritoneal lavage was obtained by washing with phosphate-buffered saline (PBS) and incubation with an anti-CD16/32 antibody (1 μg/10^6^ cells, 20 min) to block the Fc receptor. Subsequently, the peritoneal cells were incubated with biotinylated anti-CD45R/B220 (0.25 μg/10^6^ cells) and anti-CD3 (0.125 μg/10^6^ cells) antibodies for 15 min and then with streptavidin-coated microbeads (10 μl/10^7^ cells, 15 min). The mixtures were applied to a magnetic-activated cell sorting system to collect the flow-through fraction using an autoMACS Pro Separator (Miltenyi Biotec).

### RNA-seq

Total RNA was isolated using TRIzol reagent (Invitrogen). RNA quality was assessed using the Agilent 2100 Bioanalyzer (Agilent Technologies), and the RNA was quantified using an ND-2000 Spectrophotometer (Thermo). Libraries were prepared from the total RNA using the NEBNext Ultra II Directional RNA-Seq Kit (New England BioLabs). mRNA was isolated using the Poly(A) RNA Selection Kit (Lexogen) and was used for cDNA synthesis and shearing, following the manufacturer’s instructions. Indexing was performed using Illumina indices 1–12. The libraries were enriched using PCR. Fragment size was evaluated using the Agilent 2100 Bioanalyzer (DNA High Sensitivity Kit). Quantification was performed using a library quantification kit and a StepOne RT‒PCR System (Life Technologies). High-throughput sequencing was performed as paired-end 100 sequencing using a NovaSeq 6000 instrument (Illumina).

### RNA-seq data analysis

Raw sequencing data were quality-controlled using FastQC. Adapter and low-quality reads ( < Q20) were removed using FASTX_Trimmer^23^ and BBMap^24^. The trimmed reads were mapped to the reference genome using TopHat^25^. Gene expression levels were estimated based on fragments per kilobase per million reads values using Cufflinks^26^. The fragments per kilobase per million reads values were normalized based on quantile normalization using the EdgeR package within R^27^. Data mining and graphic visualization were performed using ExDEGA (Ebiogen).

### ChIP analysis

Cells were washed twice with PBS and fixed in 1% formaldehyde, and fixation was quenched by the addition of 125 mM glycine. After the cells were harvested, they were lysed with 0.5% NP-40-containing buffer A (5 mM PIPES at pH 8.0 and 85 mM KCl) and sonicated using a Bioruptor (low power, Diagenode). After the removal of cell debris by centrifugation at 14,000 rpm for 15 min, the cell lysates were sonicated using a Bioruptor (high power) to shear the DNA to fragments of 200–400 base pairs. After preclearing with protein G Dynabeads, the appropriate antibody was added, and the cells were incubated at 4 °C overnight. The next day, antibody-chromatin complexes were captured with protein G Dynabeads and washed with low- and high-salt wash buffers and LiCl wash buffer. The chromatin antibody immobilized on the magnetic beads was then subjected to tagmentation. Eluted DNA was purified using the MinElute kit (Qiagen) and subjected to RT‒qPCR analysis.

### Immunofluorescence assay

Cells were grown on coverslips in 24-well plates. Following treatments, the cells were fixed using 4% formaldehyde and permeabilized using 0.2% Triton X-100. After blocking with 4% bovine serum albumin, the cells were incubated with anti-LAMP1 (1:250), anti-C/EBPβ (1:250), and anti-cathepsin B antibodies (1:200) followed by incubation with Cy3-conjugated anti-rat and mouse IgG and Alexa Fluor 488-conjugated anti-goat IgG antibodies. F-actin was stained using rhodamine-phalloidin (Thermo), and nuclei were visualized by counterstaining with DAPI. Images were acquired using a confocal microscope (LSM 700, Zeiss) and processed using ZEN 3.5 software.

### Statistical analyses

All values are expressed as the mean ± standard error of the mean (SEM). All data were pooled from at least three independent experiments. The exact *n* number of independent experiments is described in each figure legend. Data were analyzed using one-way analysis of variance followed by Dunnett’s *post hoc* tests for comparison of all groups with the control group, two-way analysis of variance with Bonferroni *post hoc* correction for comparison between control and *Bmal1*-deficient mouse groups, or Student’s *t* tests. The level of statistical significance was set at *p* ≤ 0.05. Analyses were performed using GraphPad Prism 5.

## Results

### *Bmal1* deficiency increases noncanonical inflammasome-mediated pyroptosis in macrophages

**Fig. 1:**
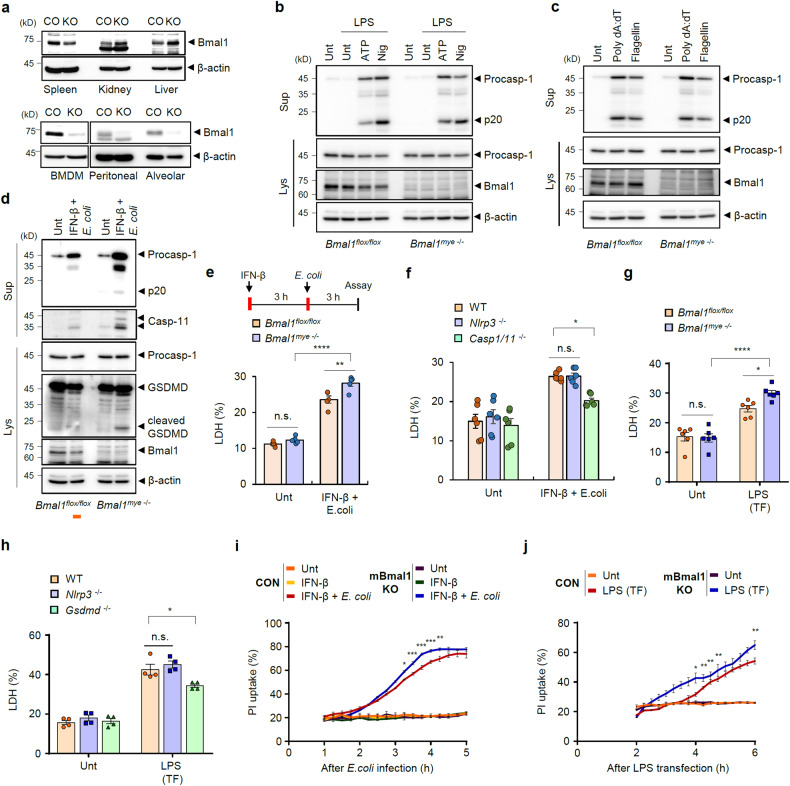
Myeloid *Bmal1* deficiency increases noncanonical inflammasome-mediated pyroptosis. **a** Immunoblots of tissue extracts or BMDMs, peritoneal macrophages and alveolar macrophages from control (CO, *Bmal1*^flox/flox^) and myeloid-specific *Bmal1*-KO mice. **b** Representative immunoblots of *Bmal1*^flox/flox^ and myeloid *Bmal1*^–/–^ BMDMs untreated (Unt) or treated with LPS (100 ng/ml, 3 h) alone or followed by ATP (3 mM, 1 h) or nigericin (2.5 μM, 1 h) treatment. (*n* = 3). **c** Representative immunoblots of control and myeloid *Bmal1*-deficient BMDMs transfected with poly(dA:dT) (1 μg/ml, 2 h) or flagellin (250 ng/ml, 2 h). (*n* = 3). **d**, **e** Representative immunoblots (**d**) and quantification of LDH in culture supernatants (**e**) of *Bmal1*^flox/flox^ and myeloid *Bmal1*^–/–^ BMDMs treated with IFN-β (500 pg/ml, 3 h) followed by *E. coli* infection (MOI 20, 3 h). (**d**, *n* = 3; **e**, *n* = 5) (**f**) Quantification of LDH release from WT, *Nlrp3*-deficient, and *Casp-1/11*-deficient BMDMs treated as in (**e**). (*n* = 6). **g**, **h** Quantification of LDH release by *Bmal1*^flox/flox^ and myeloid *Bmal1*^–/–^ BMDMs (**g**) or WT (*Bmal1*^+/+^), *Nlrp3*-deficient, and *Gsdmd*-deficient BMDMs (**h**) 3 h post transfection of LPS (2 μg/ml). (**g**, *n* = 6; **h**, *n* = 4). **i** Quantification of relative PI uptake by control and myeloid *Bmal1*-KO BMDMs treated with IFN-β, followed by *E. coli* infection in the presence of PI (10 μg/ml). Fluorescence was measured every 15 min. (*n* = 3). **j** Quantification of relative PI uptake by control and myeloid *Bmal1*-KO BMDMs 2 h after LPS transfection. (*n* = 3) Culture supernatants (Sup) or cellular lysates (Lys) were immunoblotted with the indicated antibodies (**b**–**d**). Data represent the mean ± SEM. *P < 0.05, **P < 0.01, ***P < 0.001, ****P < 0.0001, n.s. not significant.

To investigate whether circadian clock deficiency in macrophages affects the sensitivity of the inflammasome response, we generated myeloid-specific *Bmal1*-knockout (KO) mice. BMAL1 expression was effectively abolished in bone marrow-derived macrophages (BMDMs) as well as peritoneal or alveolar macrophages but not in peripheral organs, such as the spleen, kidneys, and liver, from myeloid *Bmal1*-deficient mice (*LysM*^*cre/+*^ *Bmal1*^*flox/flox*^) (Fig. 1a). We next examined the inflammasome-activating potential of BMDMs from control (*Bmal1*^*flox/flox*^) and *Bmal1*-deficient mice (*Bmal1*^*mye -/-*^) by evaluating the presence of active interleukin (IL)-1β or caspase-1 in culture supernatants. *Bmal1*-deficient macrophages exhibited similar levels of IL-1β secretion (Supplementary Fig. 1a) and caspase-1 cleavage (Fig. 1b and Supplementary Fig. 1b) as control macrophages in response to NLRP3 inflammasome-activating stimulation (lipopolysaccharide (LPS) + ATP or nigericin). Similarly, there was no significant difference in caspase-1 processing between control and *Bmal1*-KO BMDMs in response to poly(dA:dT) (AIM2 inflammasome agonist) or flagellin (NLRC4 inflammasome agonist) transfection (Fig. 1c and Supplementary Fig. 1c). These data demonstrate that *Bmal1* deficiency does not significantly alter the responsiveness of macrophages to canonical NLRP3-, NLRC4-, and AIM2-mediated inflammasome signaling at the cellular level.

We then examined the noncanonical inflammasome response in macrophages stimulated with interferon (IFN)-β priming and subsequent *E. coli* infection. Intriguingly, *Bmal1* deficiency caused a marked increase in caspase-11 secretion and gasdermin D (GSDMD) cleavage in macrophages in response to IFN-β-*E. coli* stimulation (Fig. 1d). Consistent with this finding, *Bmal1*-deficient BMDMs showed significantly increased release of lactic acid dehydrogenase (LDH), a typical pyroptosis indicator^28^, upon IFN-β-*E. coli* stimulation compared to the control cells (Fig. 1e). IFN-β priming and *E. coli* infection caused caspase-1/-11-dependent pyroptosis (Fig. 1f), indicating that these stimulants trigger caspase-11-mediated noncanonical inflammasome signaling. Supporting these observations, *Bmal1*-deficient macrophages exhibited enhanced LDH release by *E. coli* infection, which also induced caspase-1/-11-dependent cell death (Supplementary Fig. 2a, b). However, there was no difference in canonical inflammasome-mediated pyroptosis between control and *Bmal1*-deficient BMDMs (Supplementary Fig. 2c, d).

To validate the relevance of *Bmal1* deficiency in noncanonical inflammasome-mediated pyroptosis, we examined intracellular LPS-induced pyroptosis in both types of macrophages using LPS transfection. Intracellular LPS-driven LDH release was significantly increased in the *Bmal1*-deficient BMDMs compared to the control cells (Fig. 1g). LPS transfection-mediated cell death was dependent on GSDMD, a crucial molecule of pyroptosis^29^ (Fig. 1h). Then, we further confirmed the increased pyroptosis in *Bmal1*-deficient macrophages by measuring the uptake of propidium iodide (PI). Consistently, myeloid *Bmal1*-KO BMDMs exhibited significantly increased PI uptake after IFN-β-*E. coli* stimulation (Fig. 1i) or LPS transfection (Fig. 1j). These results demonstrate that *Bmal1* deficiency leads to an enhanced noncanonical inflammasome response and pyroptosis in macrophages.

### Myeloid *Bmal1* deficiency increases mouse lethality in response to noncanonical inflammasome stimulation

**Fig. 2:**
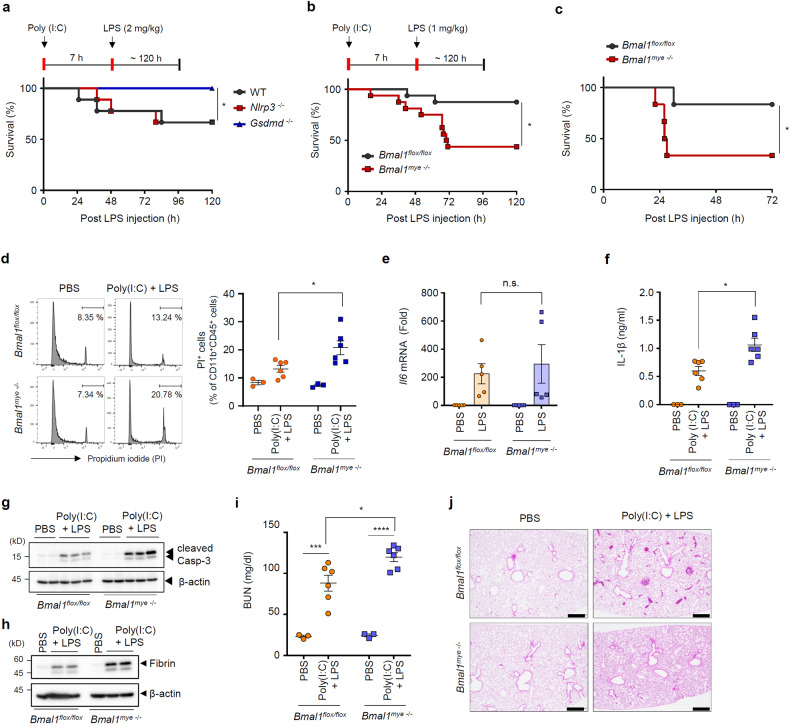
Myeloid *Bmal1* deficiency increases noncanonical inflammasome-mediated mouse lethality. **a** Survival of WT, *Nlrp3*^–/–^, and *Gsdmd*^–/–^ mice after intraperitoneal injection of poly(I:C) (10 mg/kg, 7 h) and subsequent administration of LPS (2 mg/kg). (*n* = 12; male 4, female 8). **b** Survival of *Bmal1*^flox/flox^ and myeloid *Bmal1*^–/–^ mice intraperitoneally injected with poly(I:C), followed by LPS (1 mg/kg) injection. (*n* = 16; male 5, female 11). **c** Survival of *Bmal1*^flox/flox^ and myeloid *Bmal1*^–/–^ mice after intraperitoneal injection of LPS (20 mg/kg) (*n* = 6; male). **d** Flow cytometric analysis of peritoneal cells from *Bmal1*^*flox/flox*^ and myeloid *Bmal1*^*–/–*^ mice intraperitoneally injected with poly(I:C) for 7 h and challenged with LPS (1 mg/kg) for 4 h after staining with anti-CD45 and CD11b antibodies and propidium iodide (PI). (PBS, *n* = 3; poly(I:C) + LPS, *n* = 6). **e** Quantification of *Il6* mRNA levels in *Bmal1*^flox/flox^ and myeloid *Bmal1*^–/–^ BMDMs at 3 h post LPS treatment (100 ng/ml) (*n* = 5). **f** Quantification of IL-1β in serum from *Bmal1*^flox/flox^ and myeloid *Bmal1*^–/–^ mice intraperitoneally injected with poly(I:C) for 7 h and challenged with LPS (1 mg/kg) for 3 h. (PBS, *n* = 3; poly(I:C) + LPS, *n* = 6). **g**, **h** Immunoblots of spleen extracts from *Bmal1*^*flox/flox*^ and myeloid *Bmal1*^*–/–*^ mice intraperitoneally injected with poly(I:C) for 7 h and challenged with LPS (1 mg/kg) for 14 h (**g**) or 24 h (**h**). (*n* = 3). **i** Quantification of blood urea nitrogen (BUN) levels in serum from *Bmal1*^flox/flox^ and myeloid *Bmal1*^–/–^ mice intraperitoneally injected with poly(I:C) for 7 h and challenged with LPS (1 mg/kg) for 24 h. (PBS, *n* = 3; poly(I:C) + LPS, *n* = 6). **j** Representative hematoxylin and eosin staining images of *Bmal1*^flox/flox^ and myeloid *Bmal1*^–/–^ mice treated as in (**h**). (*n* = 4) Scale bar = 500 μM. Data represent the mean ± SEM. *P < 0.05, ***P < 0.001, ****P < 0.0001, n.s. not significant.

Next, we assessed the in vivo susceptibility of control and myeloid *Bmal1*-deficient mice to noncanonical inflammasome activation. To induce noncanonical inflammasome activation in vivo, mice were given an intraperitoneal injection of poly(I:C), a Toll-like receptor 3 (TLR3) agonist, and a low dose of LPS (1 or 2 mg/kg). Poly(I:C) priming and subsequent LPS administration caused *Gsdmd*-dependent pyroptosis-mediated lethality in mice (Fig. 2a). Notably, poly(I:C)-LPS-induced mouse lethality was significantly increased by myeloid *Bmal1* deficiency (Fig. 2b). Consistent herewith, *Bmal1*-deficient mice were more susceptible to a high dose of LPS challenge (20 mg/kg) (Fig. 2c), which also induced *Gsdmd*-dependent lethality (Supplementary Fig. 3a). To examine whether poly(I:C)-LPS-induced mouse lethality is mediated by myeloid pyroptosis, we assessed our in vivo noncanonical inflammasome-mediated sepsis model in myeloid-specific *Gsdmd*-deficient mice. Consequently, mouse lethality induced by poly(I:C)-LPS was completely abolished in the myeloid *Gsdmd*-deficient mice (Supplementary Fig. 3b). Furthermore, poly(I:C) priming, followed by LPS stimulation, caused elevated myeloid cell death in the peritoneum of *Bmal1*-deficient mice compared to control mice (Fig. 2d). These results indicate that myeloid circadian clock BMAL1 is closely implicated in the susceptibility of mice to noncanonical inflammasome activation.

We next evaluated the potential role of BMAL1 in the innate immune response in vitro by stimulating BMDMs with LPS or poly(I:C). *Bmal1* deficiency did not significantly alter LPS-induced IL-6 production in BMDMs (Fig. 2e and Supplementary Fig. 4a). Likewise, treatment with poly(I:C) induced similar levels of type I IFN production in the *Bmal1*-deficient and control BMDMs (Supplementary Fig. 4b). Furthermore, there was no significant difference in retinoic acid-inducible gene I-mediated IFN-β production induced by poly(I:C) transfection between control and *Bmal1*-KO macrophages (Supplementary Fig. 4c).

Next, we examined the phenotypes of control and myeloid *Bmal1*-deficient mice after poly(I:C) + LPS administration. *Bmal1* ablation caused an increase in the serum level of IL-1β, but not IL-6, upon poly(I:C) + LPS challenge (Fig. 2f and Supplementary Fig. 4d). Poly(I:C) priming with LPS administration enhanced caspase-3 cleavage in the spleen more strongly in the *Bmal1*-deficient mice than in the control mice (Fig. 2g), indicating that *Bmal1* deficiency caused more severe spleen damage upon noncanonical inflammasome activation. Increased blood clotting as determined by fibrin or prothrombin was proposed as a *Gsdmd*-dependent septic lethality^30^. Consistently, we found an elevation of fibrin levels in the spleen of *Bmal1*-deficient mice. (Fig. 2h). Consistent with these findings, *Bmal1*-deficient mice exhibited more severe kidney and lung damage upon poly(I:C) + LPS administration than control mice (Fig. 2i, j). These results demonstrate that myeloid *Bmal1* deficiency increases the susceptibility of mice to noncanonical inflammasome-mediated organ damage and pyroptosis-dependent lethality.

### *Bmal1* deficiency leads to reduced expression of Rev-erbα in mouse macrophages

**Fig. 3:**
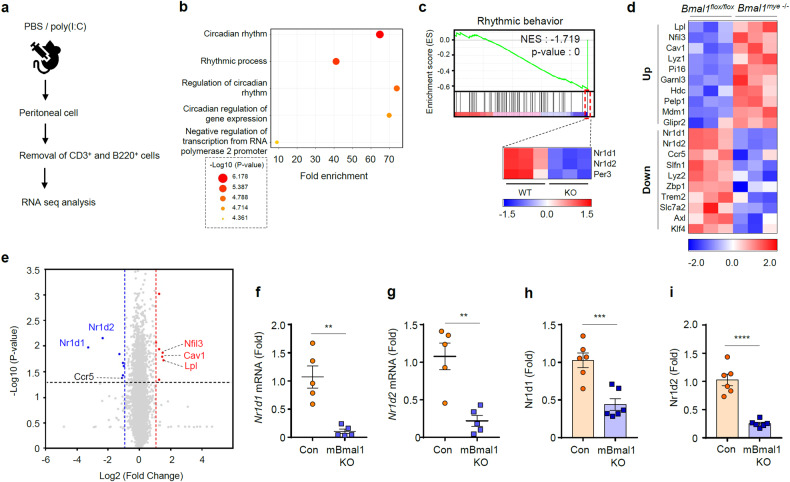
Myeloid Bmal1 deficiency leads to the decreased expression of Nr1d1 and Nr1d2. **a** Experimental scheme for myeloid cell enrichment from peritoneal cells of mice. **b** Bubble chart of the top five GO terms enriched in genes in peritoneal myeloid cells from myeloid *Bmal1*-KO mice compared to those from control (*Bmal1*^flox/flox^) mice ( > 2-fold, *P* ≤ 0.05). **c** GSEA plot of the rhythmic behavior pathway and heatmap of the major genes in this pathway. Heatmap (**d**) and volcano plot (**e**) of differentially expressed genes in peritoneal myeloid cells from myeloid *Bmal1*^–/–^ mice compared to *Bmal1*^flox/flox^ mice ( > 1.5-fold or <1.5-fold and *P* ≤ 0.05). Quantification of *Nr1d1* (**f**) and *Nr1d2* (**g**) mRNA levels in peritoneal myeloid cells from control and myeloid *Bmal1-*KO mice (*n* = 5). Quantification of *Nr1d1* (**h**) and *Nr1d2* (**i**) mRNA levels in *Bmal1*^*flox/flox*^ and myeloid *Bmal1*^*–/–*^ BMDMs (*n* = 6). Data represent the mean ± SEM. **P < 0.01, ***P < 0.001, ****P < 0.0001.

To elucidate the molecular mechanism by which myeloid cell-specific deletion of *Bmal1* increases mouse lethality upon noncanonical inflammasome stimulation, we performed RNA-sequencing (RNA-seq) analysis to comprehensively compare gene expression levels in peritoneal myeloid cells isolated from control and *Bmal1*-deficient mice. Prior to the analysis, we enriched myeloid cells from isolated peritoneal cells by removing T (CD3^+^) and B (B220^+^) cells through cell-specific antibody-mediated magnetic-activated cell sorting (Fig. 3a and Supplementary Fig. 5).

Gene Ontology (GO) analysis of genes with a more than 2-fold change in expression after *Bmal1* KO (*P* < 0.05) revealed that myeloid *Bmal1* ablation caused robust changes in the expression of circadian rhythm-related genes (Fig. 3b). To further characterize the effect of *Bmal1* deletion on the transcriptome, we performed gene set enrichment analysis (GSEA) using Kyoto Encyclopedia of Genes and Genomes (KEGG). The analysis revealed that the rhythmic behavior pathway was negatively regulated by *Bmal1* KO; in particular, *Nr1d1*, *Nr1d2*, and *Per3* were significantly decreased in the *Bmal1*-KO peritoneal myeloid cells (Fig. 3c). Based on the RNA-seq data, we generated a heatmap of the top 10 up- and downregulated genes in *Bmal1*-deficient versus control peritoneal myeloid cells (Fig. 3d) and a volcano plot of the differentially expressed genes (Fig. 3e). We then validated the expression of selected highly up- and downregulated candidate genes in peritoneal cells using RT‒qPCR analysis. The expression levels of *Lpl* and *Nfil3* were significantly elevated in peritoneal myeloid cells of the myeloid *Bmal1*-deficient mice (Supplementary Fig. 6a, b). However, their expression levels in the *Bmal1*-deficient BMDMs were not significantly different from those in the control cells (Supplementary Fig. 6c, d). In contrast, the expression levels of *Nr1d1* and *Nr1d2*, encoding Rev-erbα and Rev-erbβ, were decreased in mouse peritoneal myeloid cells (Fig. 3f, g) and BMDMs (Fig. 3h, i) of the myeloid *Bmal1*-deficient mice. These findings indicate that *Bmal1* deficiency leads to a significant reduction in the expression of *Nr1d1* and *Nr1d2* in myeloid cells at steady state.

### *Bmal1* deficiency leads to increased expression of SAA1 in response to poly(I:C) stimulation

**Fig. 4:**
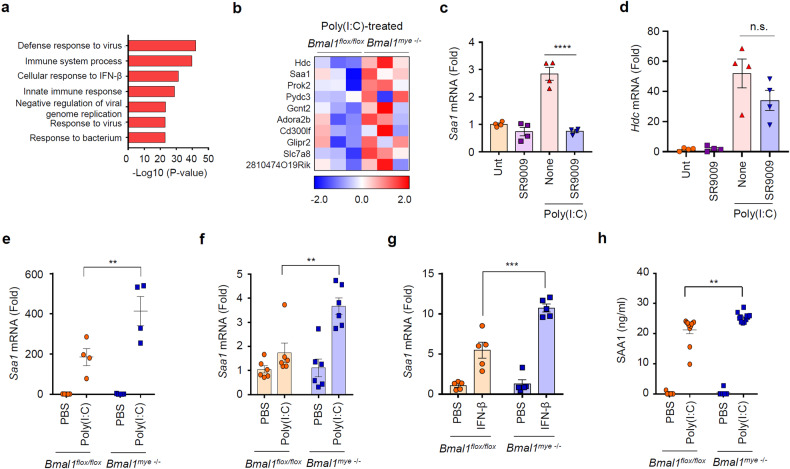
*Saa1* is upregulated in myeloid Bmal1-deficienct macrophages upon poly(I:C) stimulation. **a** GO analysis of 397 genes that exhibit significant upregulation in peritoneal myeloid cells from both control and myeloid *Bmal1*-KO mice following poly(I:C) stimulation, in comparison to mice treated with PBS ( > 5-fold and *P* ≤ 0.05). **b** Heatmap of genes significantly upregulated in peritoneal myeloid cells from poly(I:C)-challenged myeloid *Bmal1*-KO mice compared with poly(I:C)-challenged control mice ( > 1.25 fold and *P* ≤ 0.05). Quantification of *Saa1* (**c**) and *Hdc* (**d**) mRNA levels in BMDMs (from WT mice) treated with SR9009 (10 μM, 3 h) followed by treatment with poly(I:C) (5 μg/ml, 6 h) (*n* = 4). **e** Quantification of *Saa1* mRNA levels in peritoneal myeloid cells from *Bmal1*^*flox/flox*^ and myeloid *Bmal1*^*–/–*^ mice intraperitoneally injected with PBS or poly(I:C) (10 mg/kg, 3 h). (*n* = 4). **f**, **g** Quantification of *Saa1* mRNA levels in *Bmal1*^*flox/flox*^ and myeloid *Bmal1*^*–/–*^ BMDMs treated with poly(I:C) (5 μg/ml, 3 h) (*n* = 6, f) or IFN-β (500 pg/ml, 2 h) (*n* = 5, **g**). **h** Quantification of SAA1 levels in peritoneal lavage from *Bmal1*^*flox/flox*^ and myeloid *Bmal1*^*–/–*^ mice intraperitoneally injected with PBS or poly(I:C) (10 mg/kg, 6 h). (PBS, *n* = 7; poly(I:C), *n* = 11) Data represent the mean ± SEM. **P < 0.01, ***P < 0.001, ****P < 0.0001, n.s. not significant.

To provide molecular insight into the increased lethality of *Bmal1*-deficient mice upon poly(I:C) + LPS challenge, we profiled gene expression in peritoneal myeloid cells from control and *Bmal1*-deficient mice after poly(I:C) stimulation. Poly(I:C) injection caused a significant upregulation ( > 5-fold, *P* < 0.05) of 397 genes in peritoneal myeloid cells from mice of both groups. GO analysis of these 397 genes revealed that genes related to antiviral and antibacterial responses were mainly increased in response to poly(I:C) administration (Fig. 4a). Furthermore, we sorted 397 genes by their expression in the poly(I:C)-treated *Bmal1*-deficient versus control myeloid cells (Fig. 4b). Among the top five upregulated genes in *Bmal1*-deficient cells, we examined whether their increased expression is correlated with *Nr1d1* expression using the Rev-erbα agonist SR9009 because Rev-erbα was remarkably downregulated in the *Bmal1*-deficient macrophages in our RNA-seq analysis. Notably, poly(I:C)-induced expression of *Saa1* mRNA was significantly reduced by SR9009 (Fig. 4c), while the expression of other top upregulated genes, including *Hbc*, *Prok2*, *Pydc3* and Gcn2, was not impaired by SR9009 (Fig. 4d and Supplementary Fig. 7). These data indicate that Rev-erbα activity is potentially implicated in the poly(I:C)-induced expression of *Saa1*, which encodes serum amyloid A1 (SAA1), a major acute phase protein in the regulation of the inflammatory process^31^. We then validated the mRNA expression of *Saa1* in wild-type and *Bmal1*-deficient macrophages. Consistent with the transcriptome analysis, *Saa1* mRNA expression was significantly increased in the peritoneal myeloid cells of myeloid Bmal1 KO mice compared to control mice upon poly(I:C) stimulation (Fig. 4e). Supporting these findings, *Bmal1*-deficient macrophages exhibited a significant increase in *Saa1* mRNA levels in BMDMs upon poly(I:C) (Fig. 4f) or IFN-β stimulation (Fig. 4g) compared to control cells. In addition, myeloid *Bmal1*-KO mice exhibited a significant increase in SAA1 levels in peritoneal lavage fluid (Fig. 4h) compared to control mice in response to poly(I:C) administration. These data demonstrate that *Bmal1* deficiency leads to increased SAA1 production in myeloid cells of mice upon poly(I:C) or IFN-β treatment.

### SAA1 increases noncanonical inflammasome-mediated pyroptosis and mouse lethality

**Fig. 5:**
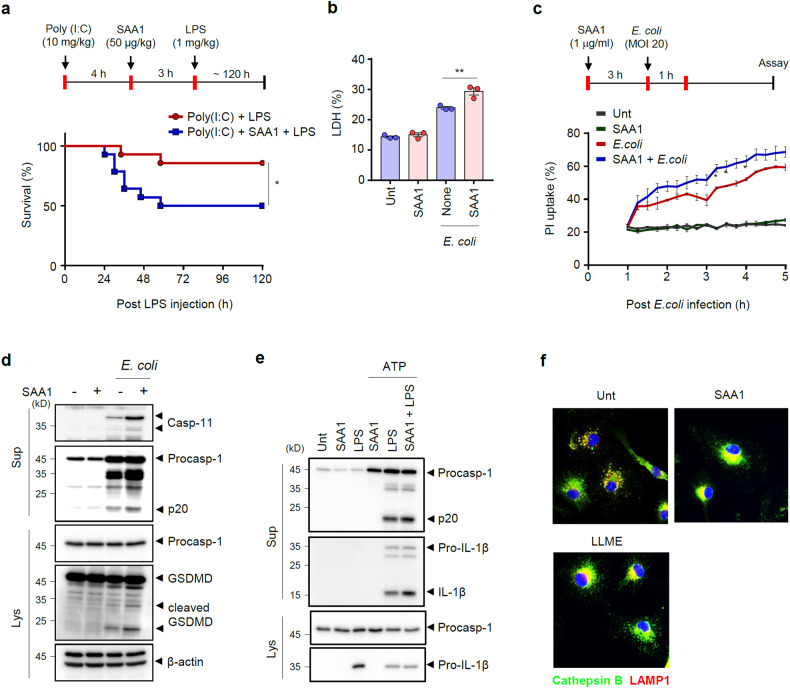
SAA1 increases noncanonical inflammasome-mediated lethality and pyroptosis. **a** Survival of mice intraperitoneally injected with poly(I:C) (10 mg/kg) and challenged with LPS (1 mg/kg) in the presence or absence of SAA1 (50 μg/kg). (*n* = 14; female). **b** Quantification of LDH in culture supernatants of BMDMs treated with SAA1 (1 μg/ml, 3 h) alone or followed by *E. coli* infection (MOI 20, 2 h) (*n* = 3). **c** Quantification of PI uptake by BMDMs treated with SAA1 (1 μg/ml, 3 h), followed by *E. coli* infection (MOI 20, 1 h), in the presence of PI (*n* = 3). **d** Representative immunoblot of BMDMs treated with SAA1 (1 μg/ml, 3 h), followed by *E. coli* infection (MOI 20, 2 h) (*n* = 3). **e** Representative immunoblot of BMDMs treated with SAA1 (1 μg/ml, 3 h) or LPS (100 ng/ml, 3 h), followed by ATP treatment (3 mM, 1 h) (*n* = 3). **f** Representative immunofluorescence images of BMDMs treated with SAA1 (1 μg/ml) and LLME (0.4 mM) for 3 h. (*n* = 3) Bar = 20 μM. Data represent the mean ± SEM. *P < 0.05, **P < 0.01.

To examine the potential role of SAA1 in the noncanonical inflammasome-mediated response, we administered recombinant SAA1 to mice during poly(I:C) and LPS injection. Notably, SAA1 administration enhanced mouse lethality induced by poly(I:C) + LPS challenge (Fig. 5a). Since poly(I:C) stimulation caused a notable elevation in SAA1 in the peritoneal lavage of myeloid *Bmal1*-deficient mice (Fig. 4h), we assessed systemic SAA1 levels in mice under noncanonical inflammasome-mediated septic conditions. In contrast to the increased SAA1 levels in the peritoneum, there was no significant difference in SAA1 production levels in the serum and liver between control and myeloid *Bmal1*-deficient mice following poly(I:C) + LPS administration (Supplementary Fig. 8a, b). These findings suggest that the elevated production of SAA1 in the peritoneum of *Bmal1*-deficient mice may contribute to the increased lethality. In accordance with mouse lethality, *E. coli* infection-induced pyroptosis of macrophages was significantly elevated by the addition of SAA1 (Fig. 5b, c), indicating that SAA1 augments the intensity of noncanonical inflammasome response. In support of these results, we found that exogenous SAA1 potentiated the secretion of caspase-11 and GSDMD cleavage from BMDMs upon *E. coli* infection (Fig. 5d). However, exogenous addition of SAA1 did not enhance LPS/ATP-induced canonical inflammasome activation, as shown by the processing and secretion of caspase-1 and IL-1β in macrophages (Fig. 5e). Instead, SAA1 treatment caused robust lysosome destabilization in macrophages, as did the lysosomotropic agent l-leucyl-l-leucine methyl ester (LLME) (Fig. 5f). At steady state, the luminal lysosomal protease cathepsin B colocalized with LAMP1, a lysosomal membrane protein. However, treatment with SAA1 or LLME caused lysosomal membrane rupture, as shown by the dispersion of cathepsin B in the cytosol and the disruption of colocalization with LAMP1. These results collectively demonstrate that SAA1 treatment increases pyroptosis in macrophages and mouse lethality upon noncanonical inflammasome-activating stimulation.

### Rev-erbα regulates poly(I:C)-induced SAA1 expression by inhibiting CEBPβ

**Fig. 6:**
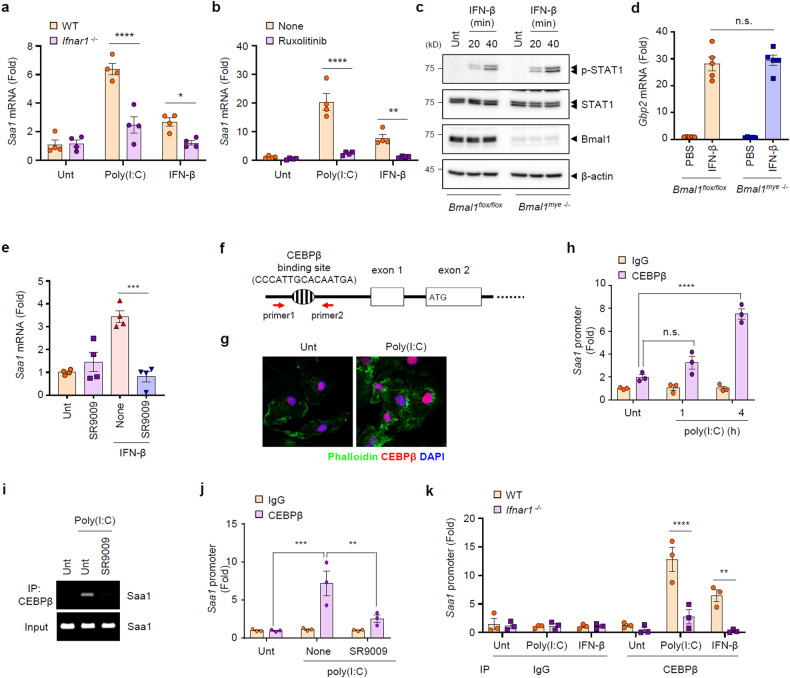
Rev-erbα negatively regulates poly(I:C)-induced *Saa1* mRNA production through inhibition of CEBPβ activation. **a** Quantification of *Saa1* mRNA levels in WT and *Ifnar1*^*-/-*^ BMDMs treated with poly(I:C) (5 μg/mL, 6 h) or IFN-β (500 pg/ml, 2 h) (*n* = 4). **b** Quantification of *Saa1* mRNA levels in BMDMs treated with ruxolitinib (500 nM, 1 h) followed by treatment with poly(I:C) (5 μg/mL, 6 h) or IFN-β (500 pg/ml, 2 h) (*n* = 4). **c** Immunoblot of *Bmal1*^*flox/flox*^ and myeloid *Bmal1*^*–/–*^ BMDMs treated with IFN-β (500 pg/ml, 20 or 40 min) (*n* = 3). **d** Quantification of *Gbp2* mRNA levels in *Bmal1*^*flox/flox*^ and myeloid *Bmal1*^*–/–*^ BMDMs treated with IFN-β (500 pg/ml, 2 h) (*n* = 5). **e** Quantification of *Saa1* mRNA levels in BMDMs (from WT mice) treated with SR9009 (10 μM, 3 h) followed by treatment with IFN-β (500 pg/ml, 2 h) (*n* = 4). **f** Nucleotide sequences of upstream enhancer elements of the mouse *Saa1* promoter and primer design for ChIP-PCR and RT-qPCR. **g** Representative immunofluorescence images of BMDMs treated with poly(I:C) (5 μg/ml, 6 h). Bar = 20 μM. **h** ChIP-qPCR analysis of BMDMs treated with poly(I:C) (5 μg/ml, 1 or 4 h) using anti-CEBPβ or anti-IgG antibodies (*n* = 3). **i**, **j** ChIP-PCR (**i**) and RT-qPCR (**j**) analyses of BMDMs treated with SR9009 (10 μM, 3 h) followed by treatment with poly(I:C) (5 μg/ml, 4 h), using anti-CEBPβ or anti-IgG antibodies (*n* = 3). **k** ChIP-qPCR analysis of WT and *Ifnar1-/-* BMDMs treated with poly(I:C) (5 μg/ml, 4 h) or IFN-β (500 pg/ml, 1 h) using anti-CEBPβ or anti-IgG antibodies (*n* = 3). Data represent the mean ± SEM. *P < 0.05, **P < 0.01, ***P < 0.001, ****P < 0.0001, n.s. not significant.

To elucidate the mechanism by which *Bmal1* deletion facilitates the upregulation of SAA1, we evaluated the potential relevance of type 1 IFN receptor signaling for SAA1 expression because IFNβ-induced SAA1 expression was stronger in the *Bmal1*-deficient macrophages than in the control cells (Fig. 4g). Of note, the ablation of the IFN receptor significantly impaired poly(I:C)- and IFNβ-stimulated *Saa1* mRNA induction (Fig. 6a). Supporting this finding, the JAK1 inhibitor ruxolitinib markedly suppressed *Saa1* expression (Fig. 6b) as well as STAT1 phosphorylation by IFNβ (Supplementary Fig. 11a). These observations indicate that poly(I:C)-mediated SAA1 expression is a downstream event of type 1 IFN receptor signaling. Then, we examined a potential difference in type 1 IFN receptor-mediated signaling between control and *Bmal1*-deficient macrophages. STAT1 phosphorylation following IFNβ treatment exhibited a similar level regardless of *Bmal1* expression (Fig. 6c). Consistent with this finding, there was no significant difference in *Gbp2* mRNA expression, an interferon-stimulated gene (ISG), between control and *Bmal1*-deficient macrophages (Fig. 6d), indicating that *Bmal1* deficiency did not affect conventional type 1 IFN receptor signaling events.

Instead, we observed that Rev-erbα plays a specific role in regulating *Saa1* mRNA expression in response to poly(I:C) stimulation, as evidenced by the use of Rev-erbα agonist SR9009 (Fig. 4c). Consistently, SR9009 significantly inhibited IFN-β-stimulated Saa1 expression (Fig. 6e). However, SR9009 did not reduce poly(I:C)- or IFN-β-promoted mRNA expression of IL-6 and IFN-β in BMDMs (Supplementary Fig. 9a–c). These results suggest that Rev-erbα is a direct regulator of poly(I:C)- or IFN-β-induced SAA1 production and that Rev-erbα-mediated SAA1 expression is different from conventional TLR and type 1 IFN receptor signaling.

In this context, we searched for a potential transcription factor for SAA1 expression and found a C/EBPβ-binding site in the promotor region of *Saa1* (Fig. 6f). Our RNA-seq analysis revealed that mouse peritoneal myeloid cells express high levels of *Cebpb* (Supplementary Fig. 10a). In BMDMs, poly(I:C) stimulation led to a slight but significant increase in C/EBPβ expression (Supplementary Fig. 10b). C/EBPβ was clearly detected in the nucleus of BMDMs, regardless of poly(I:C) stimulation (Fig. 6g). Intriguingly, chromatin immunoprecipitation (ChIP) analysis revealed that poly(I:C) stimulation (4 h) induced robust binding of C/EBPβ to the promoter region of *Saa1* in BMDMs (Fig. 6h). This binding was significantly impaired by the Rev-erbα agonist SR9009 (Fig. 6i, j), indicating that Rev-erbα potentially inhibits poly(I:C)-induced association of C/EBPβ with the *Saa1* promoter region. Furthermore, type 1 IFN receptor deficiency significantly attenuated C/EBPβ-*Saa1* promoter binding induced by poly(I:C) or IFNβ stimulation (Fig. 6k). These results demonstrate that type 1 IFN receptor signaling is essential for C/EBPβ-mediated SAA1 induction in a Rev-erbα-dependent manner. Supporting these findings, the inhibition of JAK1, a downstream molecule of type 1 IFN receptor, markedly reduced *Saa1* expression (Fig. 6b) and noncanonical inflammasome-mediated pyroptosis (Supplementary Fig. 11b).

### Inhibition of Rev-erbα increases poly(I:C)-induced SAA1 production and noncanonical inflammasome-mediated pyroptosis and mouse lethality

**Fig. 7:**
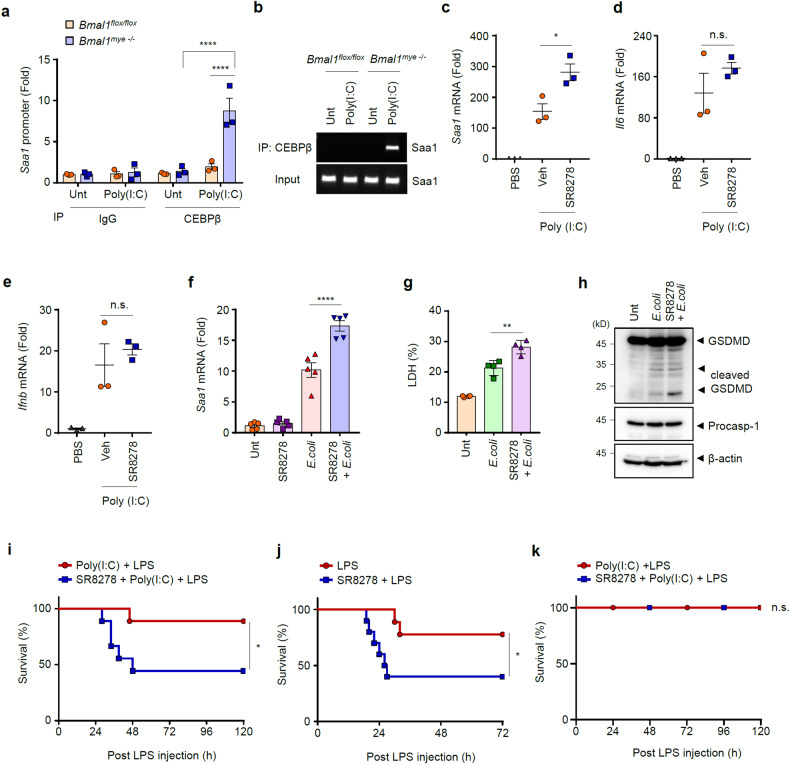
Rev-erbα decreases sepsis lethality by inhibiting CEBPβ-mediated Saa1 production. ChIP-qPCR (**a**) and PCR (**b**) analyses of *Bmal1*^flox/flox^ and myeloid *Bmal1*^–/–^ BMDMs treated with poly(I:C) (5 μg/ml, 1 h), using anti-CEBPβ or anti-IgG antibody (*n* = 3). Quantification of *Saa1* (**c**), *Il6* (**d**), and *Ifnb* (**e**) mRNA levels in peritoneal myeloid cells from mice intraperitoneally injected with PBS or poly(I:C) (10 mg/kg, 3 h) in the presence or absence of SR8276 (20 mg/kg, 3 h) (*n* = 3). **f**, **g** Quantification of *Saa1* (**f**) mRNA and extracellular LDH (**g**) of BMDMs treated with SR8278 (20 μM, 3 h) followed by *E. coli* infection (MOI 20, 2 h) (*n* = 5). **h** Representative immunoblots of cellular lysates from BMDMs treated with SR8278 (20 μM, 3 h) followed by *E. coli* infection (MOI 20, 2 h) (*n* = 3). **i** Survival of mice intraperitoneally injected with poly(I:C) (10 mg/kg, 7 h) in the presence or absence of SR8278 (20 mg/kg, 3 h) and challenged with LPS (1 mg/kg). (*n* = 9; female). **j** Survival of mice intraperitoneally injected with LPS (20 mg/kg) in the presence or absence of SR8278 (20 mg/kg, 3 h) (*n* = 9; female). **k** Survival of myeloid *Gsdmd*^*-/-*^ mice intraperitoneally injected with poly(I:C) (10 mg/kg, 7 h) in the presence or absence of SR8278 (20 mg/kg, 3 h) and challenged with LPS (1 mg/kg). (*n* = 8; male 4, female 4) Data represent the mean ± SEM. *P < 0.05, **P < 0.01, ****P < 0.001, n.s. not significant.

Consistent with the above data, *Bmal1*-deficient macrophages, which expressed substantially lower levels of Rev-erbα than control cells, showed greatly enhanced binding of C/EBPβ with the *Saa1* promoter in response to 1 h of poly(I:C) treatment (Fig. 7a, b). We then examined the physiological relevance of Rev-erbα activity in poly(I:C)-induced SAA1 production using SR8278, a Rev-erbα antagonist. Similar to *Bmal1*-deficient mice, SR8278-treated mice exhibited significantly increased *Saa1* mRNA levels in peritoneal myeloid cells upon poly(I:C) stimulation (Fig. 7c). However, SR8278 administration did not alter poly(I:C)-induced *Il6* and *Ifnb* mRNA levels in peritoneal myeloid cells (Fig. 7d, e). To further confirm whether the blockade of Rev-erbα by SR8278 could regulate noncanonical inflammasome-mediated SAA1 production and pyroptosis in macrophages, we examined the potential effect of SR8278 treatment in BMDMs upon *E. coli* infection. Consequently, SR8278 treatment caused a significant increase in *E. coli*-induced *Saa1* mRNA production (Fig. 7f) and pyroptosis (Fig. 7g, h).

Finally, we assessed whether inhibition of Rev-erbα would affect the in vivo susceptibility of mice to a noncanonical inflammasome agonist. Notably, SR8278 administration led to a significant reduction in mouse survival after poly(I:C) + LPS challenge (Fig. 7i). In support of this finding, SR8278-mediated blockade of Rev-erbα increased mouse lethality induced by a high dose of LPS, which also triggered noncanonical inflammasome signaling (Fig. 7j). In contrast, SR8278 treatment did not enhance poly(I:C)-LPS-induced lethality in myeloid *Gsdmd*-deficient mice (Fig. 7k), indicating that Rev-erbα can affect myeloid pyroptosis-mediated mouse lethality. These results demonstrate that Rev-erbα is a key regulator of the noncanonical inflammasome response and consequent mouse lethality.

## Discussion

Circadian clock-dependent immune regulation is an interesting phenomenon. Numerous immune parameters, including the numbers of circulating cells and serum cytokine levels, display daily and circadian oscillations^32^. In particular, deletion of circadian clock genes in myeloid cells strongly enhanced the innate immune response and susceptibility to septic shock^12,13^. Furthermore, the expression of clock-associated genes was downregulated in septic shock patients^33^. However, the involvement of the circadian clock in inflammasome-mediated responses remains unclear. Here, we found that myeloid-specific *Bmal1* deletion did not alter the canonical inflammasome response in macrophages but significantly increased noncanonical inflammasome-mediated pyroptosis in macrophages and mouse lethality. Recent studies have demonstrated that pyroptosis following caspase-11-mediated noncanonical inflammasome activation is closely related to the mortality rate and the pathogenesis of endotoxin-mediated sepsis^20,29^. However, the correlation between circadian disruption and pyroptosis has not yet been studied. Our findings reveal that the circadian clock modulates the sensitivity of macrophages to pyroptosis in a stimulation-dependent manner.

Although previous studies have demonstrated that circadian rhythm disruption enhances the severity of inflammatory disorders, such as septic shock^13^, asthma^34^, atherosclerosis^17^, and experimental autoimmune encephalomyelitis^14^, the underlying mechanisms remain poorly understood. Furthermore, a recent study demonstrated that myeloid-specific deletion of *Bmal1* or the *clock* gene did not increase the endotoxin lethality of mice kept in constant darkness^35^. In this context, our results demonstrate that *Bmal1* deficiency causes a downregulation of Rev-erbα, which can block C/EBPβ to induce SAA1 production, leading to an enhanced noncanonical inflammasome response (Supplementary Fig. 12). Rev-erbα has been recently shown to modulate target molecules in a circadian rhythm-dependent manner^36^. C/EBPβ is a key transcription factor for the induction of SAA1^37^. However, the circadian-dependent regulation of SAA1 expression remains unclear. In this regard, our data indicate that stimulation-dependent SAA1 expression can be regulated via the Bmal1-Rev-erbα circadian clock. Intriguingly, our results also revealed that type 1 IFN receptor signaling is needed for C/EBPβ-*Saa1* promoter binding, which is further modulated by Rev-erbα. Considering that there was no significant difference in IFNβ-stimulated ISG expression between control and *Bmal1*-deficient cells, *Saa1* induction operates distinctively from conventional type 1 IFN signaling and is instead controlled by Rev-erbα. Further investigations will be needed to clarify this aspect. Collectively, C/EBPβ and SAA1 may be crucial players in the propagation of pyroptosis under the control of the circadian clock.

Rev-erbα is a transcriptional repressor that inhibits the expression of not only *Bmal1* but also other cell- or tissue-specific genes, including inflammatory cytokine genes^38,39^. Recent studies have demonstrated that Rev-erbα deficiency worsens various inflammatory diseases, such as hepatitis^40^, colitis^41^, and neuroinflammation^42^. These studies have suggested that Rev-erbα can block NF-κB signaling, leading to a reduction in the expression of diverse target genes. Of interest, recent studies have demonstrated that Rev-erbα negatively regulates the expression of NLRP3 inflammasome components, such as NLRP3, Il-1β, and Il-18^40,41^. In our study, Rev-erbα levels were substantially diminished in the *Bmal1*-deficient macrophages, which also showed slightly increased NLRP3 expression compared to that in the control cells. A recent study demonstrated that Bmal1-deficient macrophages exhibited enhanced expression of pro-IL-1β compared to control cells^43^. However, *Bmal1* deficiency did not alter NLRP3 inflammasome activation itself. These data indicate that the circadian arrhythmia-mediated increase in the expression of inflammasome components does not lead to NLRP3 inflammasome activation.

Inflammasome activation triggers the maturation of the proinflammatory cytokines IL-1β and IL-18 and the induction of pyroptosis, an inflammatory caspase-mediated lytic cell death^44,45^. Pyroptosis is induced by not only the canonical caspase-1 inflammasome pathway but also the noncanonical caspase-4/5/11 (caspase-4/5 in humans and caspase-11 in mice)-mediated inflammasome pathway^46,47^. Cytoplasmic LPS can directly bind to and activate caspase-11, independent of TLR4, and subsequently promote GSDMD-dependent pyroptosis^20^. Recent studies have suggested that pyroptosis through cytosolic LPS-mediated caspase-11 activation is a key feature of sepsis-mediated mortality in patients^20,29^. Although further elucidation of how pyroptosis of myeloid cells can induce systemic lethality is needed, recent findings have demonstrated that pyroptosis-dependent release of tissue factors from macrophages triggers systemic blood clotting, leading to host death^30^. The physiological role of the circadian clock in caspase-11-mediated noncanonical inflammasome signaling has not yet been reported. Our findings reveal that *Bmal1* deficiency enhances caspase-11-mediated pyroptosis in macrophages. Therefore, circadian disruption, which frequently occurs in people with insomnia and nightwork, can increase susceptibility to the bacterial infection-mediated noncanonical inflammasome response and pyroptosis.

SAA1 has been identified as a biomarker in ulcerative colitis and sepsis^48,49^. SAA1 is a major acute-phase protein mainly produced in the liver, but it is also expressed in other cell types, including endothelial cells, smooth muscle cells, and macrophages, in response to infection and tissue injury^50^. During sepsis, SAA1 is highly induced in the acute phase^51^, and extracellular SAA1 stimulates various innate immune receptors, including TLR2^52^, TLR4^53^, and NLRP3^54^. Furthermore, SAA1 can bind directly to LPS to form a complex that facilitates the intracellular localization of LPS^55^. Once internalized into macrophages, SAA1 causes lysosomal destabilization through the formation of SAA1 fibrils^56^. However, the role of SAA1 in the inflammasome response and pyroptosis has not been elucidated. In the present study, SAA1 was significantly upregulated in *Bmal1*-deficient macrophages upon poly(I:C) stimulation. More intriguingly, exogenous SAA1 exacerbated noncanonical inflammasome-mediated sepsis, pathogenesis, and lethality. Collectively, our findings indicate that inflammation-driven SAA1 plays a key role in lethal sepsis pathogenesis by promoting caspase-11-mediated pyroptosis. However, it remains unclear how SAA1 increases sensitivity to the noncanonical inflammasome response and pyroptosis. Factors other than SAA1 might also contribute to the increased noncanonical inflammasome response in *Bmal1*-deficient cells. Furthermore, we did not evaluate whether diurnal variation in noncanonical inflammasome-mediated pyroptosis depends on the time of the day at sepsis induction. It will be intriguing to further study these aspects.

In conclusion, this study provides molecular insight into the circadian regulation of noncanonical inflammasome-mediated pyroptosis. Circadian rhythm disruption in myeloid cells caused a significant reduction in Rev-erbα expression, which enhanced the C/EBPβ-mediated increase in SAA1 expression upon poly(I:C) stimulation. Then, the increased production of SAA1 facilitated enhanced noncanonical inflammasome activation and pyroptosis of macrophages. These findings shed light on the potent importance of the circadian clock in the modulation of noncanonical inflammasome signaling in myeloid cells.

### Supplementary information

Supplementary Information

## Data Availability

All data needed to evaluate the conclusions in the paper are present in the paper or the Supplementary Materials. RNA-Seq data are available through the National Center for Biotechnology Information Gene Expression Omnibus under accession number GSE222759.
