# Supplementary material for: Deficiency of circadian clock gene Bmal1 exacerbates noncanonical inflammasome-mediated pyroptosis and lethality via Rev-erbα-C/EBPβ-SAA1 axis
Source: Exp Mol Med. 2024 Feb 1;56(2):370–82. doi: 10.1038/s12276-024-01162-w (PMC10907614; doi:10.1038/s12276-024-01162-w)
Supplement: Supplementary file 1 — Supplementary Information [file 12276_2024_1162_MOESM1_ESM.pdf]

Deficiency of circadian clock gene *Bmal1* exacerbates noncanonical inflammasome-mediated pyroptosis and lethality via Rev-erbα-C/EBPβ-SAA1 axis

Do-Wan Shim,[1,2] Jun-Cheol Eo,[1,2] Saeyoung Kim,[1,2] Inhwa Hwang,[1] BoYoung Nam,[3] Jae-Eun Shin,[1,2] Seung Hyeok Han,[3] and Je-Wook Yu[1,2*]

[1]Department of Microbiology and Immunology, Institute for Immunology and Immunological Diseases, Yonsei University College of Medicine, Seoul 03722, Republic of Korea.

[2]Graduate School of Medical Science, Brain Korea 21 Project, Yonsei University College of Medicine, Seoul 03722, Republic of Korea.

[3]Department of Internal Medicine, Institute of Kidney Disease Research, Yonsei University College of Medicine, Seoul 03722, Republic of Korea.

Correspondence: Je-Wook Yu, e-mail: jewookyu@yuhs.ac

This supplementary information includes 'Supplementary Figures 1~12'

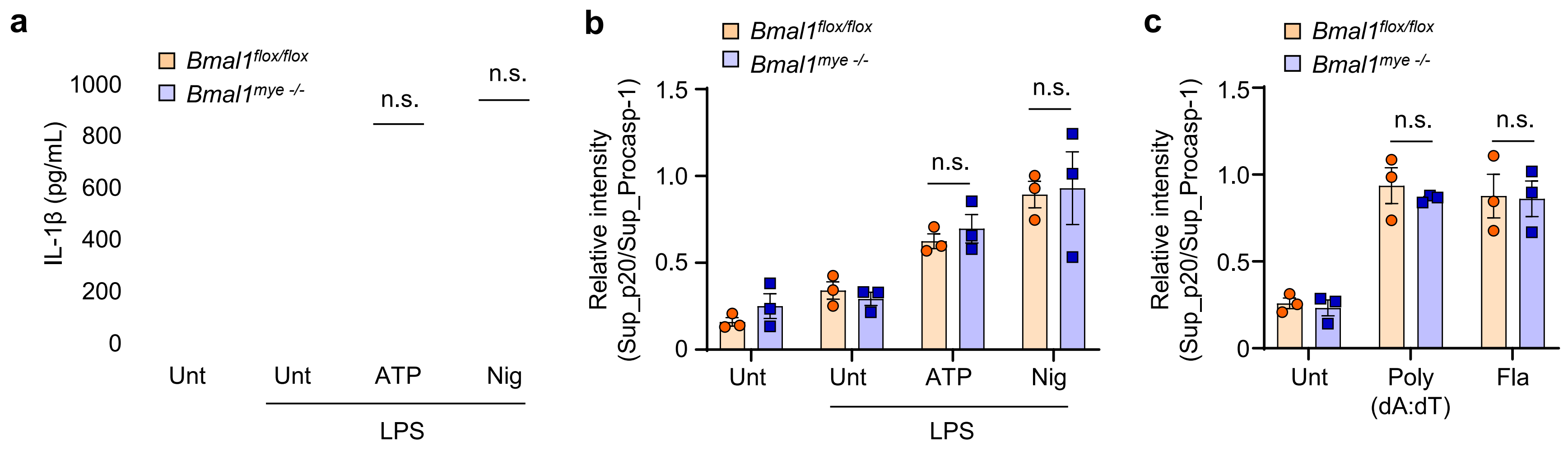

Supplementary Figure 1. *Bmal1*-deficient BMDMs exhibit similar level of canonical inflammasome response as control cells. (a) Quantification of IL-1β in culture supernatants of *Bmal1*$^{flox/flox}$ and myeloid *Bmal1*$^{-/-}$ BMDMs untreated (Unt) or treated with LPS (100 ng/ml, 3 h) alone or followed by ATP (3 mM, 1 h) or nigericin (2.5 µM, 1 h) treatment. (*n* = 3) **(b, c)** Quantification of caspase-1 p20 in the cell culture supernatant (Sup) band intensity per procaspase-1 (Sup) band intensity of Figure 1b and Figure 1c experiments. (*n* = 3) Data represent the mean ± SEM. n.s. not significant.

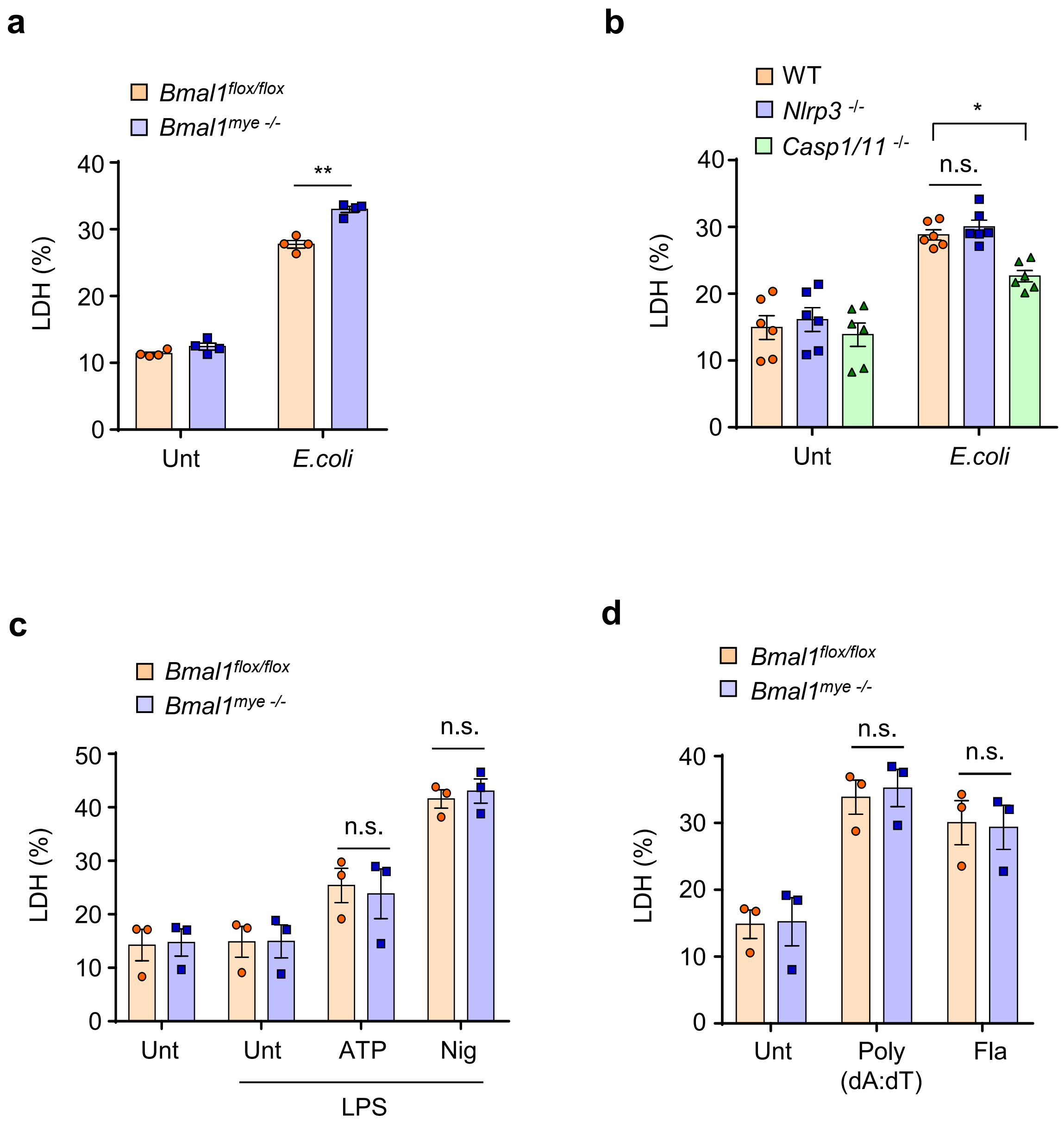

Supplementary Figure 2. *Bmal1*-deficient BMDMs exhibit increased pyroptosis in response to *E. coli* infection. (a) Quantification of LDH in culture supernatants of *Bmal1*$^{\text{flox/flox}}$ and myeloid *Bmal1*$^{-/-}$ BMDMs untreated (Unt) or infected with *E. coli* (MOI 20, 3 h). (n = 3 or 4) (b) Quantification of LDH in the culture supernatants of wild-type, *Nlrp3*-deficient or caspase-1/11-deficient BMDMs infected with *E. coli* (MOI 20, 3 h). (n = 6) (c, d) Quantification of LDH in culture supernatants of *Bmal1*$^{\text{flox/flox}}$ and myeloid *Bmal1*$^{-/-}$ BMDMs untreated (Unt) or treated with LPS (100 ng/ml, 3 h) alone or followed by ATP (3 mM, 1 h) or nigericin (2.5 µM, 1 h) treatment. (n = 3) (c), or transfected with poly(dA:dT) (1 µg/ml, 2 h) or flagellin (250 ng/ml, 2 h) using Lipofectamine 2000. (n = 3) (d) Data represent the mean ± SEM from three-independent experiments. n.s. not significant.

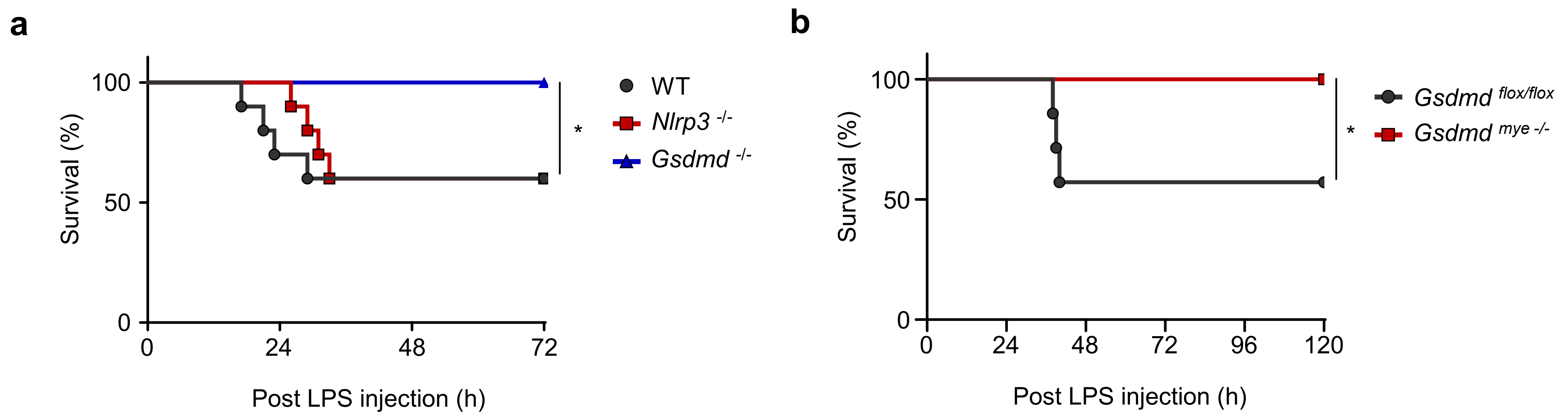

Supplementary Figure 3. GSDMD is critical for noncanonnical inflammasome-mediated septic lethality. (a) Survival of WT, *Nlrp3*$^{-/-}$, and *Gsdmd*$^{-/-}$ mice (n = 10; male 5, female 5) after intraperitoneal injection of LPS (20 mg/kg). (b) Survival of *Gsdmd*$^{flox/flox}$ (n = 7; male 3, female 4) and myeloid *Gsdmd*$^{-/-}$ (n = 8; male 4, female 4) mice intraperitoneally injected with poly(I:C), followed by LPS (1 mg/kg) injection. *$P < 0.05$.

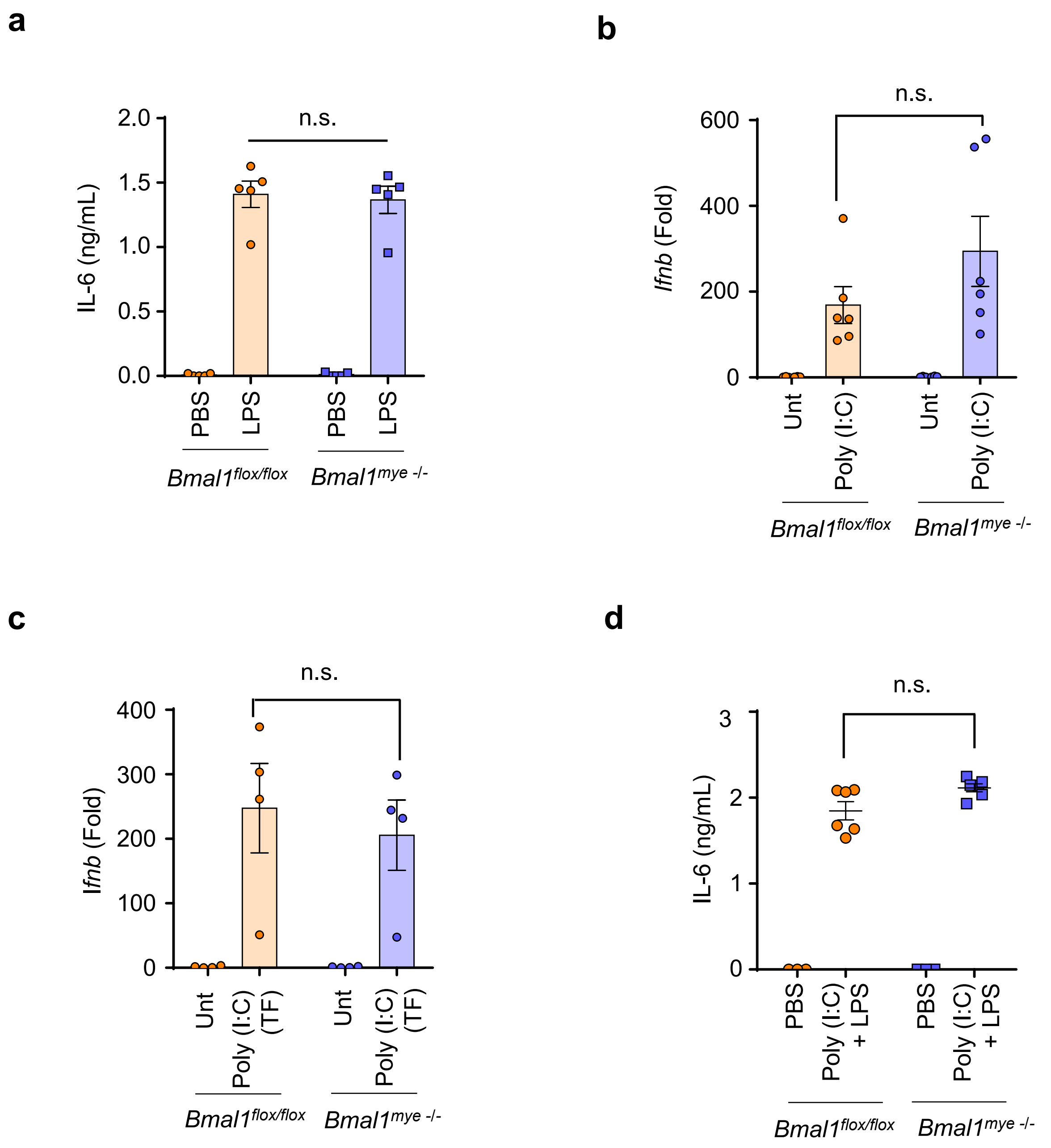

Supplementary Figure 4. *Bmal1*-deficient macrophages or mice exhibit similar pattern-recognition receptor response as control cells or mice. (a) Quantification of IL-6 in the culture supernatants of control and myeloid *Bmal1*-deficient bone marrow-derived macrophages (BMDMs) treated with LPS (100 ng/ml, 3 h). (n = 5) (b) Quantification of *Ifnb* mRNA level in control and myeloid *Bmal1*-deficient BMDMs treated with poly(I:C) (5 µg/ml, 3 h). (n = 6) (c) Quantification of *Ifnb* mRNA level in control and myeloid *Bmal1*-deficient BMDMs treated with poly(I:C) (1 µg/ml, 2 h) transfection (TF) using Lipofectamine 2000. (n = 4) (d) Quantification of IL-6 in serum from control and myeloid *Bmal1*-deficient mice intraperitoneally injected with poly(I:C) (10 mg/kg) for 7 h and then challenged with LPS (1 mg/kg) for 3 h. (PBS, n = 3; poly(I:C) + LPS, n = 6) Data represent the mean ± SEM. n.s. not significant.

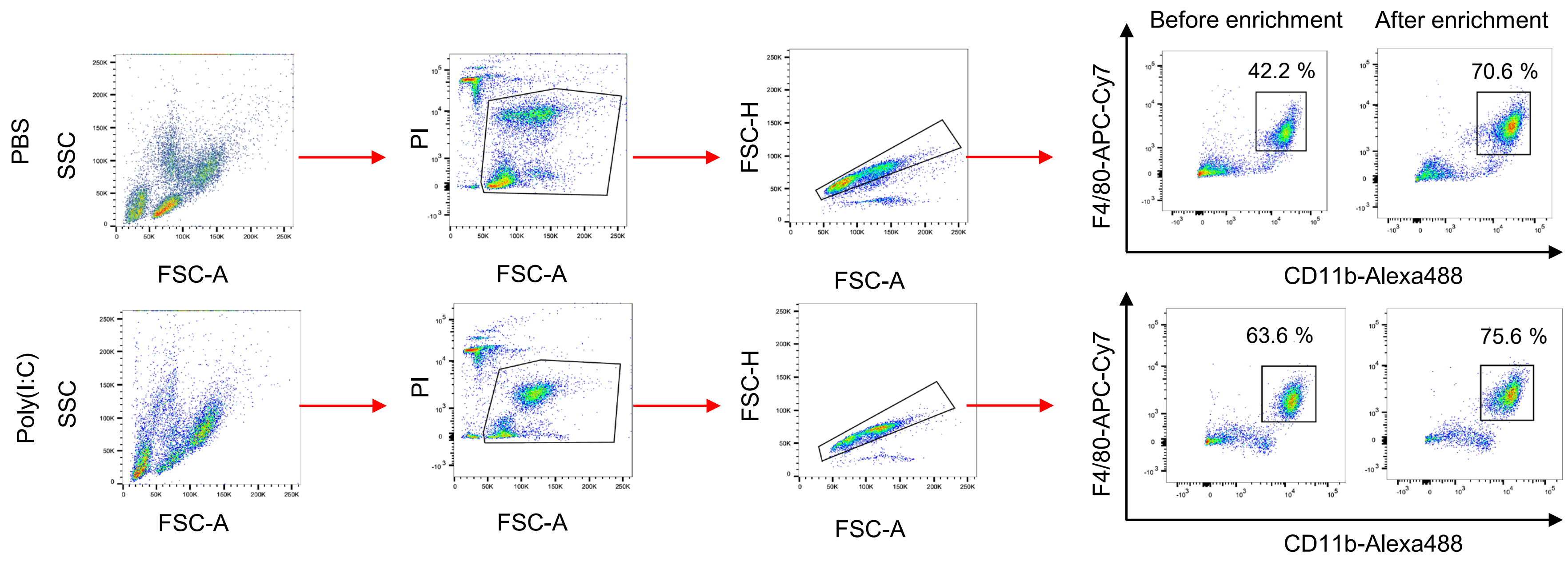

Supplementary Figure 5. Isolation of peritoneal myeloid cells. Flow cytometric analysis of peritoneal cells from mice intraperitoneally injected with PBS or poly(I:C) (10 mg/kg) for 3 h after staining with anti-CD11b and F4/80 antibody. $CD11b^{+}F4/80^{high}$ cells are peritoneal macrophage population.

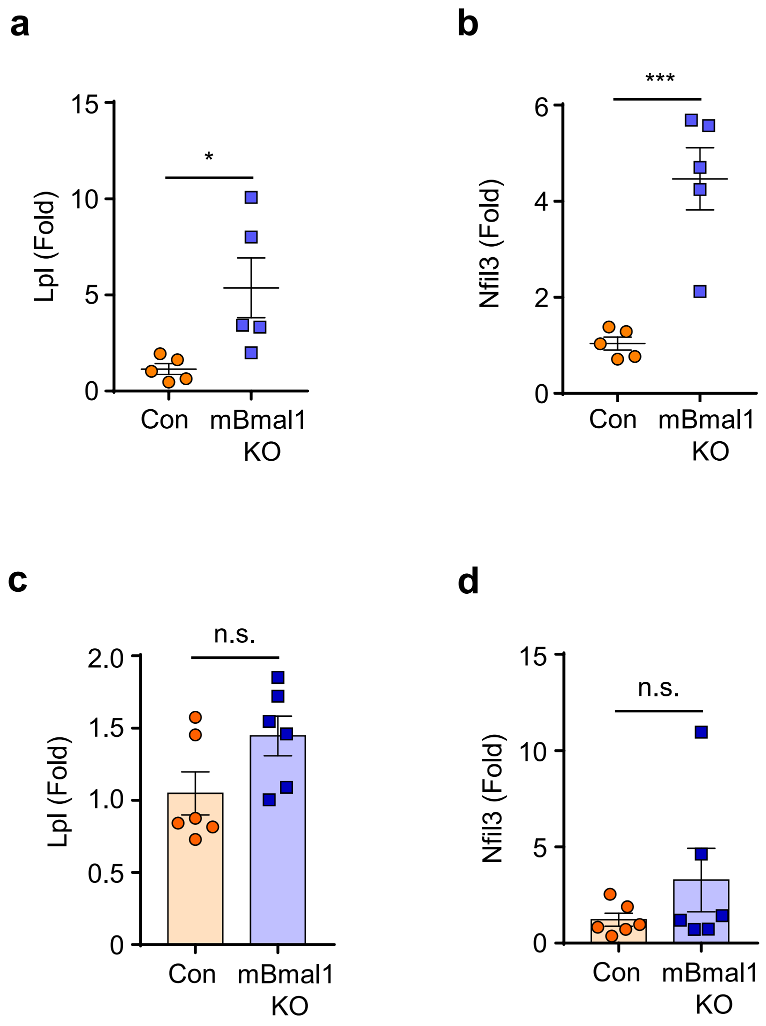

Supplementary Figure 6. Isolation of peritoneal myeloid cells and *Bmal1*-dependent changes in the gene expression of peritoneal myeloid cells and bone marrow-derived macrophages. (a and b) Quantification of *Lpl* (a) and *Nfil3* (b) mRNA level in peritoneal myeloid cells from control (con) and myeloid Bmal1 KO (knockout) mice. (n = 5) (c and d) Quantification of *Lpl* (c) and *Nfil3* (d) mRNA level in control and Bmal1 KO bone marrow-derived macrophages. (n = 6) Data represent the mean ± SEM. *$P < 0.05$, ***$P < 0.001$, n.s. not significant.

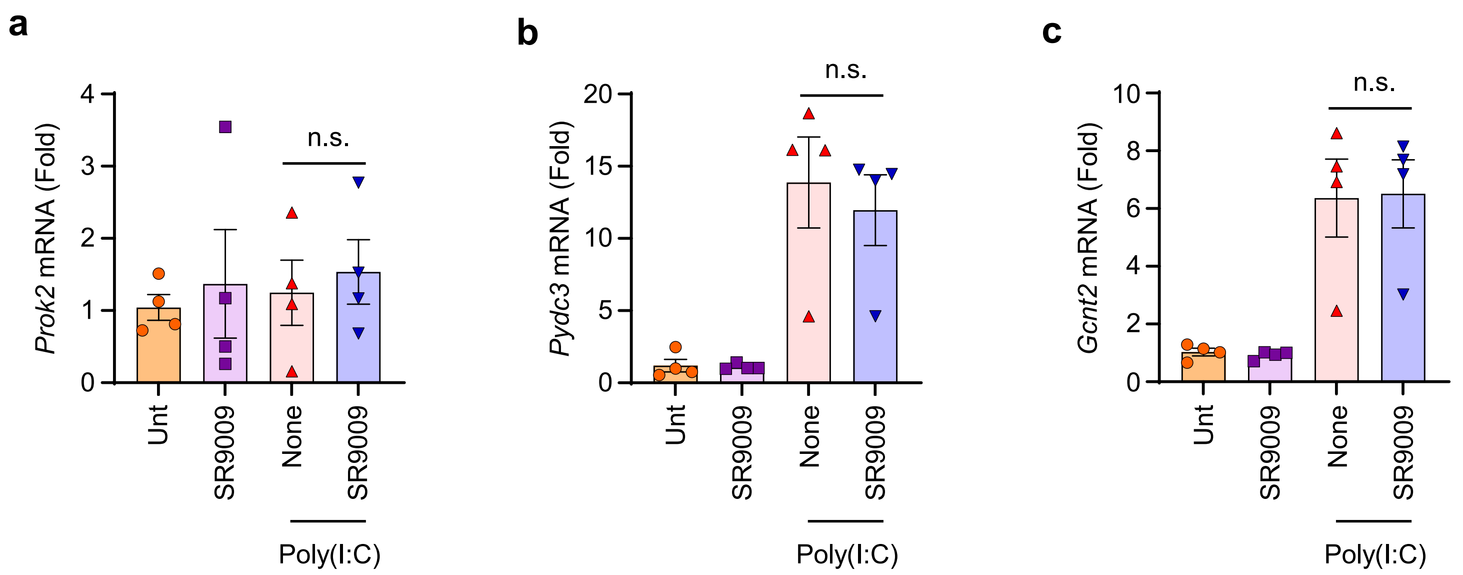

Supplementary Figure 7. Rev-erbα-dependency of poly(I:C)-induced gene expression in BMDMs. (a-c) Quantification of *Prok2* (a), *Pydc3* (b) and *Gcnt2* (c) mRNA levels in BMDMs treated with SR9009 (10 μM, 3 h) followed by treatment with poly(I:C) (5 μg/ml, 6 h). (n = 4) Data represent the mean ± SEM. n.s. not significant.

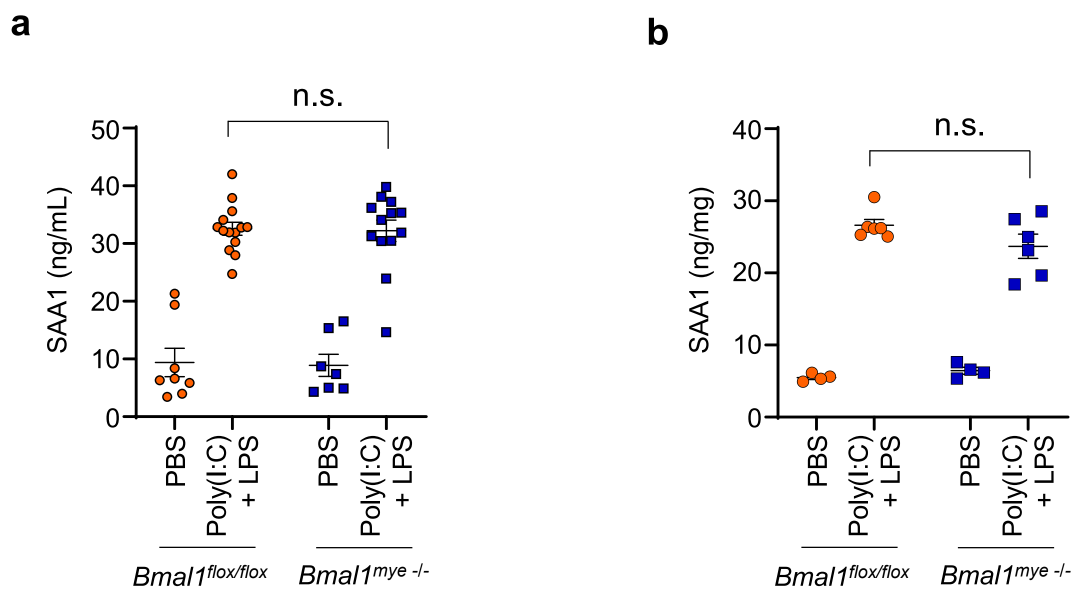

Supplementary Figure 8. Quantification of SAA1 levels in the serum or liver of mice in response to poly(I:C) and LPS stimulation. (a) Quantification of SAA1 levels in serum from *Bmal1*$^{flox/flox}$ (PBS, *n* = 8; poly(I:C)+LPS, *n* = 14) and myeloid *Bmal1*$^{-/-}$ (PBS, *n* = 7; poly(I:C)+LPS, *n* = 13) mice intraperitoneally injected with poly(I:C) (10 mg/kg) for 7 h and challenged with LPS (1 mg/kg) for 3 h. (b) Quantification of SAA1 levels in liver lysates from *Bmal1*$^{flox/flox}$ and myeloid *Bmal1*$^{-/-}$ mice intraperitoneally injected with poly(I:C) for 7 h and challenged with LPS (1 mg/kg) for 3 h. (PBS, *n* = 4; poly(I:C)+LPS, *n* = 6) Data represent the mean ± SEM. n.s. not significant.

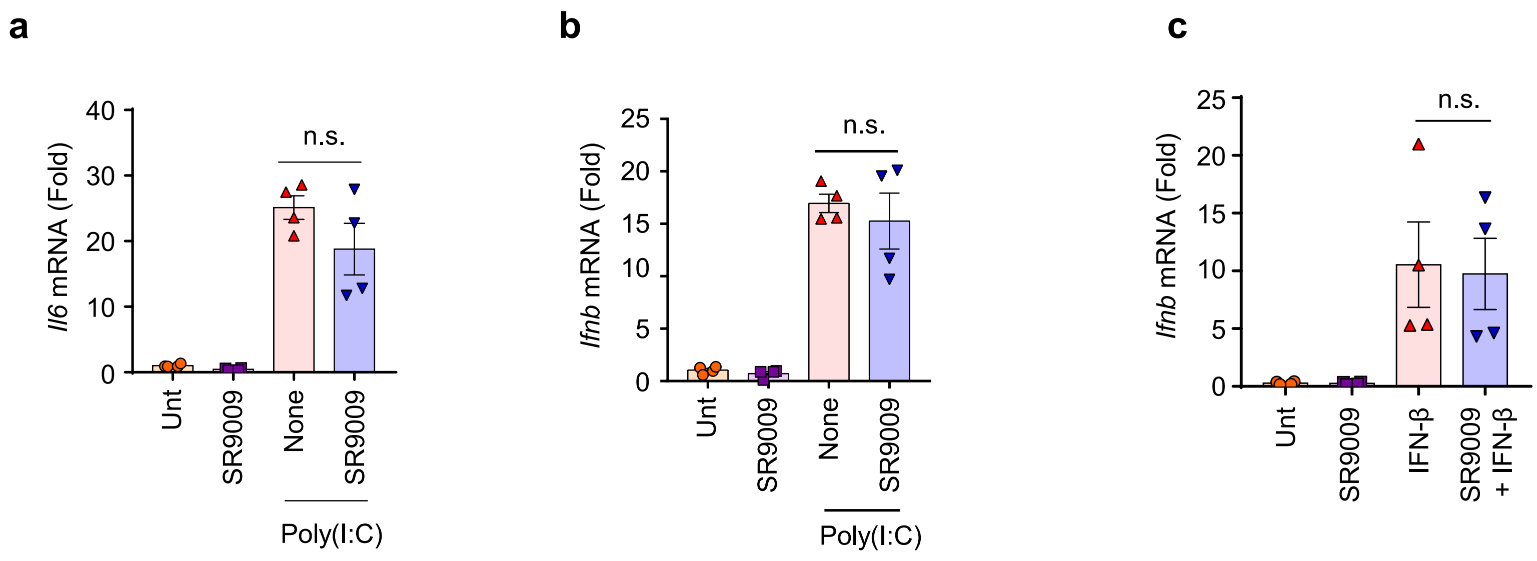

Supplementary Figure 9. SR9009 does not affect poly(I:C)- or IFN-β-stimulated *Il-6* and *Ifnb* mRNA production. (a, b) Quantification of *Il6* (a) and *Ifnb* (b) mRNA levels in BMDMs treated with SR9009 (10 μM, 3 h) followed by treatment with poly(I:C) (5 μg/ml, 6 h). (n = 4) (c) Quantification of *Ifnb* and mRNA level in BMDMs treated with SR9009 (10 μM, 3 h) followed by treatment with IFN-β (500 pg/ml, 2 h). (n = 4) Data represent the mean ± SEM. n.s. not significant.

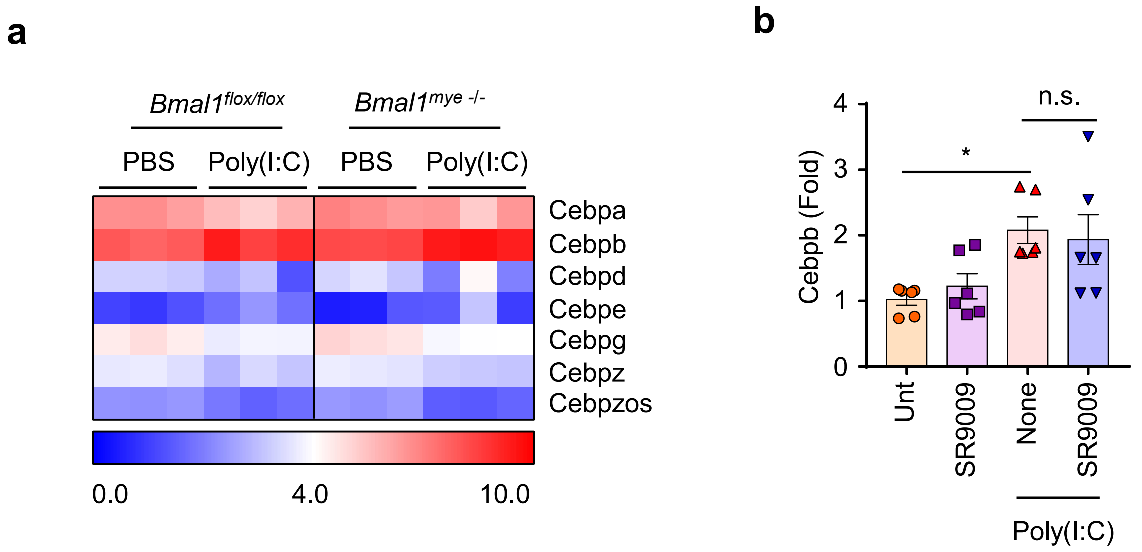

Supplementary Figure 10. Poly(I:C) stimulation induces Cebpb mRNA production. (a) Heatmap of CEBP isomers in peritoneal myeloid cells from control and myeloid *Bmal1*–deficient mice intraperitoneally injected with PBS or poly(I:C) (10 mg/kg) for 3 h. (b) Quantification of *Cebpb* mRNA expression in BMDMs treated with SR9009 (10 μM, 3 h) followed by poly(I:C) (6 h). (n = 6) Data represent the mean ± SEM. *P < 0.05, n.s. not significant.

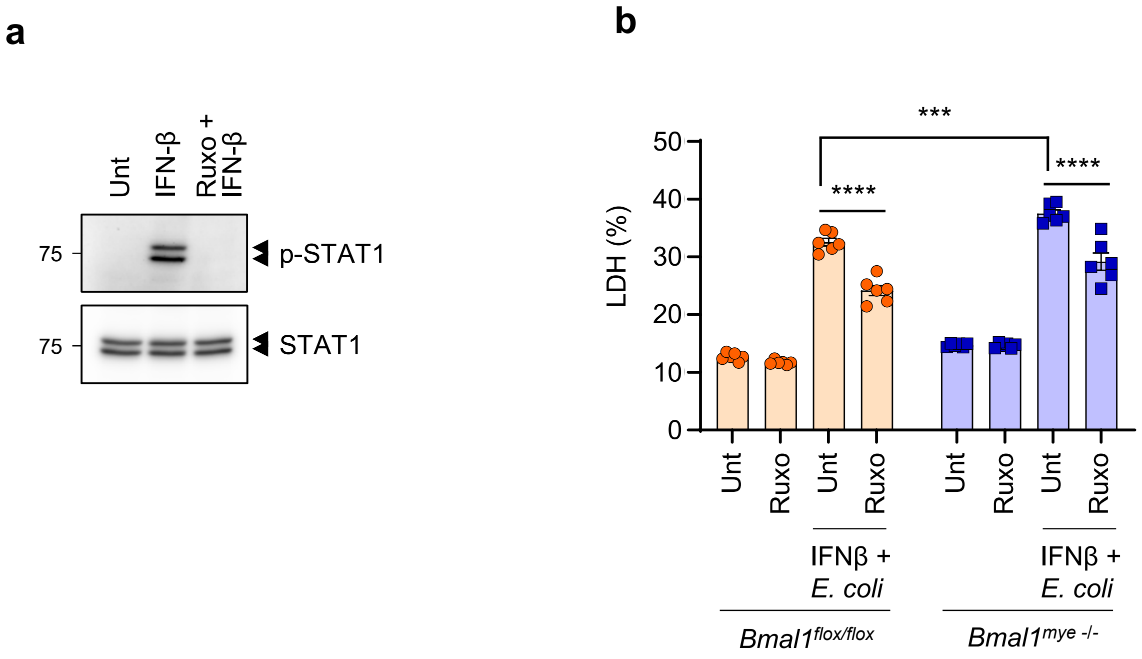

Supplementary Figure 11. Ruxolinitib inhibits noncanonical inflammasome-mediated pyroptosis. (a) Representative immunoblot of BMDMs treated with ruxolitinib (500 nM, 1 h) followed by treatment with IFN-β (500 pg/ml, 40 min). (*n* = 3) (b) Quantification of LDH in culture supernatants of *Bmal1*$^{flox/flox}$ and myeloid *Bmal1*$^{-/-}$ BMDMs treated with IFN-β (500 pg/ml, 3 h) in the presence or absence of ruxolitinib (500 nM, 1 h) followed by *E.coli* infection (MOI 20, 3 h). (*n* = 6) Data represent the mean ± SEM. ***$P < 0.001$, ****$P < 0.0001$, n.s. not significant.

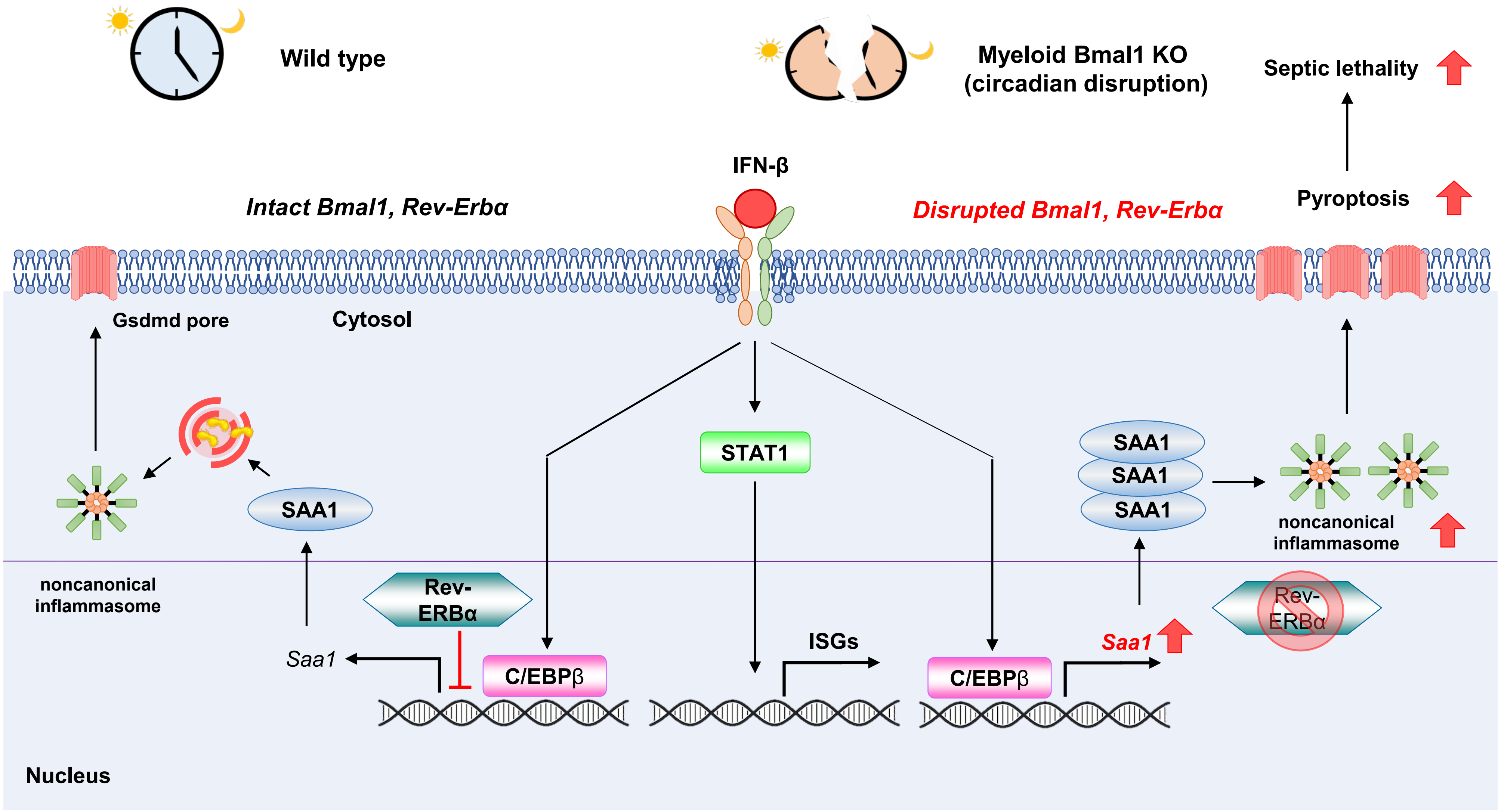

Supplementary Figure 12. Proposed mechanism how *Bmal1* deletion leads to increased noncanonical inflammasome-mediated pyroptosis. *Bmal1* deficiency caused a significant reduction of Rev-erbα, which can inhibit the association of C/EBPβ with the promoter region of *Saa1* upon poly(I:C) or interferon-β stimulation. *Bmal1* deficiency thus resulted in the increased production of SAA1, which augments noncanonical inflammasome-mediated pyroptosis.
